# Ribitol alters multiple metabolic pathways of central carbon metabolism with enhanced glycolysis: A metabolomics and transcriptomics profiling of breast cancer

**DOI:** 10.1371/journal.pone.0278711

**Published:** 2022-12-07

**Authors:** Jason Driver Tucker, Ravi Doddapaneni, Pei Juan Lu, Qi Long Lu

**Affiliations:** McColl-Lockwood Laboratory for Muscular Dystrophy Research, Atrium Health Musculoskeletal Institute, Wake Forest School of Medicine, Carolinas Medical Center, Charlotte, North Carolina, United States of America; OUHSC: The University of Oklahoma Health Sciences Center, UNITED STATES

## Abstract

Breast cancer is heterogenous in development and cell population with prognoses being highly dependent on numerous factors from driving mutations, biomarker expression and variation in extracellular environment, all affecting response to therapies. Recently, much attention has been given to the role of metabolic alteration in cancers, expanding from the Warburg effect to highlight unique patterns in different cancer cell populations for improving diagnostic and therapeutic approaches. We recently reported on modulation of mannosylation of α-dystroglycan with the metabolite ribitol in breast cancer lines. Here we investigate the effects of pentose sugars ribitol, ribose, and xylitol media supplementation in breast cancer cells by metabolomics and differential gene expression profiling. This combined approach revealed distinctive patterns of alterations in metabolic pathways by ribitol, contrasted with the closely related pentose ribose and pentitol xylitol. Significantly, ribitol supplementation enhances utilization of glucose by glycolysis, whereas ribose improves oxidative phosphorylation and fatty acid synthesis. Ribitol supplementation also increased levels of reduced glutathione (associated with a decrease in oxidative phosphorylation, gluconeogenesis), where ribose supplementation elevated levels of oxidized glutathione (GSSG) indicating an increase in oxidative stress. Treatment with ribitol also enhanced nucleotide biosynthesis. The apparent TCA cycle dysregulation, with distinctive pattern in response to the individual pentitol and pentose, such as ribitol increasing succinate and fumarate while decreasing citrate, demonstrate the adaptive capability of cancer cells to nutritional environment. This metabolic reprogramming presents new avenues for developing targeted therapies to cancers with metabolites, especially in combination with other drug treatments.

## Introduction

Cancer metabolomics is a rapidly evolving field that aims for a comprehensive dissection of the metabolic phenotypes and functional network of metabolites in human cancers [1–3]. Although the link between cancer and metabolism was initially described almost a century ago by Otto Heinrich Warburg [4], which is known as the Warburg hypothesis, substantial progress has been made after the 1990s due to the development of *in vitro* tumor models and comprehensive approaches to evaluate the metabolic status of cancer cell populations. A new era of Omics technologies leveraging high-throughput analysis of biologic molecules has revealed that alteration of cellular metabolism is one of the hallmarks of cancer [5–7], and such metabolic reprogramming occurs as a consequence of several contributing factors. These factors can be broadly classified into cell-intrinsic and -extrinsic cues [8–10]. Intrinsic cues include oncogenes and tumor suppressor genes that regulate metabolic pathways at multiple levels in different cellular compartments. Interactions between these genes and genes regulating cell proliferation and death also play critical roles. Cell-extrinsic cues comprised of nutrient availability, and interaction of the tumor cells with its microenvironment (TME) including extracellular matrix and response to status of oxygen supply (hypoxia) and changes in tissue PH (acidosis). The interplay of these factors leads cancer populations to have a greater capability for survival, growth and even metastasis. Concurrently, the reprogrammed patterns in the metabolism of cancer cells have been explored not only as hallmarks for diagnosis, but also to provide windows of opportunities for therapeutic intervention targeting particular pathways and rate-limiting substrates to counteract their advantage for survival and growth.

Recently, we have demonstrated that ribitol, a metabolite likely involved in pentose phosphate pathway (PPP) is able to enhance matriglycan levels of α-dystroglycan (α-DG) significantly and dose-dependently in the breast cancer cell line MCF7 and T47D [11]. Matriglycan-modified α-dystroglycan is expressed in all epithelial tissues including mammary glands and considered to be important for maintaining structural integrity between epithelium and extracellular matrix (ECM) through its binding to laminin and other ECM molecules [12–15]. Lack of matriglycan has been attributed to development, progression and metastasis of prostate, breast, and colorectal cancers [14, 16, 17]. Also important, the most pronounced reduction in levels of matriglycan is often observed in high-grade tumors with high proliferation index and correlated with poor prognosis [18–20]. Furthermore, enhanced expression of matriglycan is able to significantly inhibit cancer cell proliferation [20–22]. These observations support the hypothesis that alteration in the laminin-binding matriglycan plays a role in cancer development and progression and increasing expression of matriglycan could be a novel therapeutic approach for cancers. Unexpectedly, ribitol-enhanced expression of matriglycan did not inhibit proliferation of the cancer cells [11]. One possibility is that ribitol as a metabolite could alter cellular metabolism and benefit cell growth independently of matriglycan expression, alleviating inhibitory effects of ribitol-enhanced matriglycan. This hypothesis is supported by the fact that ribitol, in contrast to prior categorization as a metabolic end-product, increased the levels of ribitol-5-phosphate (ribitol-5P) and CDP-ribitol in both cancer cells and muscle cells [23], indicating that ribitol can actively participate in and likely affect cellular metabolism with yet unknown consequences. Further, tumor cell populations are known to evolve through alteration in metabolism, prominently aerobic glycolysis as well as genomic structure and gene expression, allowing cancer cells to grow and metastasize under even unfavorable microenvironments.

In the present study, we explored the role of ribitol in metabolic reprogramming of breast cancer cells using MCF7 as an *in vitro* model system by applying untargeted global metabolomics. Untargeted metabolomics has been used for probing cancer-related biochemical pathways, providing unique insight to understand changes of cancer cells under different environments and treatment regimes. Considering the fact that little is available for the possible involvement of ribitol in metabolic pathways, and to better evaluate the differential effect of ribitol and its potential significance, two widely used and chemically closely related pentose and pentitol, ribose and xylitol, were also examined for comparison. Our results demonstrate that ribitol supplementation affects a wide range of metabolic pathways with a distinctive profile in glycolysis, PPP and TCA when compared to ribose and xylitol. Ribitol supplementation enhances glycolysis with an increase in the production of pyruvate and lactate and a decrease in oxidative phosphorylation. In contrast, ribose enhanced activity of oxidative phosphorylation without clear effect on glycolysis. The differential effect of the pentitols and pentose on metabolism is further supported by gene expression profiling. The effect of ribitol on glycolysis and oxidative phosphorylation provides one possible explanation for the growth benefit observed in the cancer cells with enhanced matriglycan expression, but the potential growth advantage is not suggestive of oncogenesis as indicated by expression profiles. The unique insights of ribitol-induced reprogramming in metabolism of cancer cells could be explored for development of novel treatment strategies in combination with other drugs for breast cancer therapy.

## Materials and methods

### Tumor cell lines

The human breast cancer cell lines MCF7 (ATCC- HTB-22), T47D (ATCC-CRL2865), and MDA-MB-231 (MDA-231) (ATCC-HTB-26) were used.

MCF7 and T47D were grown in DMEM-GlutaMAX, 4.5 g/l D-glucose (10569, Gibco by life technologies) plus 10% fetal bovine serum (FBS 10082–147) and 10 μg/ml insulin (I5500 Sigma). MDA-231 were grown in DMEM-GlutaMAX+10% FBS at 37°C in a 10% CO2 incubator. The culture media were obtained from Gibco by Life Technologies. MCF-7 xenograft study in mice treated with ribitol was performed by Charles River Laboratories with IACUC approval.

### Ribitol, D-ribose, and xylitol treatment

Cells of 3x10^5^/well 6 well plate or 2x10^4^/well 96 well plate were seeded in triplicate in the growth medium unless specified otherwise. The following day, the medium was supplemented with ribitol (A5502 Sigma, St. Louis) or D-ribose (R7500 Sigma) or xylitol (X3375 Sigma) at a concentration of 10 mM, unless otherwise noted, and the cells were grown in the supplement for 3 days. Plates were washed with PBS and cells were processed for analysis by FACS, metabolomics, immunocytochemistry, and western blot.

### Global metabolomics

Non-targeted global metabolomic profiling of MCF-7 cell pellets and corresponding media was performed by Metabolon (Durham, NC, USA), according to published methods [24]. Briefly, neat methanol, containing select isotopically-labeled internal standards, was used to precipitate all the macromolecules (DNA, RNA, and protein) in the biological matrix. The purified supernatant was divided into aliquots corresponding to the various analytical methodologies, then subsequently evaporated and reconstituted with the appropriate analytical injection solvent. Samples were analyzed with four separate methods: two positive mode methods (Pos Early UHPLC-RP/MS/MS and Pos Late UHPLC-RP/MS/MS) and two negative mode methods (Neg UHPLC-RP/MS/MS and Neg UHPLC-HILIC/MS/MS) to ensure broad coverage of biochemicals.

The information output from the raw data files was automatically extracted and metabolites of known identity were recognized by comparison to metabolomic library entries of purified standards. This list of metabolites was further condensed to include only those that contained analytical values for each biological replicate, providing a total of 524 metabolites. Data are presented as fold change in three comparison groups: Untreated MCF-7 cells versus 10 mM ribitol, 10 mM Ribose, and 10 mM xylitol media-supplemented treatments.

#### Statistical analysis and pathway diagrams

Statistical analysis of log-transformed metabolomic data was performed using two-way ANOVA to assess the treatment effect. For the post-hoc contrasts, p-values and false discovery rate (FDR) were calculated according to a previously proposed method [25]. Resulting q-values were assessed across the entire dataset and significance was defined as p ≤ 0.05 and q ≤ 0.10. Data are presented as box-and-whisker plots with Tukey whiskers that show mean (+), minimum, 25% quartile, median, 75% quartile, and maximum.

### Flow cytometry

Antibody IIH6 was used to assess the level of matriglycan on α-DG. Cells were harvested by gentle cell scraper and total number counted. Cells were first blocked with 1% FBS and 1% normal goat serum in PBS for 30 minutes on ice, washed with PBS and then incubated with IIH6 at x100 dilution in PBS/0.1%FBS for 60 min on ice. After washing twice with PBS, the cells were stained with secondary antibody Alexa 594-conjugated goat-anti-mouse/IgM at x100 dilution for 45 min on ice, washed and resuspended in 500 μl FACS buffer containing 1% BSA, 0.1% NaN_3_ in PBS and analyzed by FACS. All experiments were done in triplicate.

### Immunocytochemistry (ICC)

Cell cultures for ICC were washed with PBS before fixation with ice-cold methanol for 10 minutes. Residual methanol was removed by washing with PBS and air dry. Cells were rehydrated prior to staining procedures with PBS and blocked with 6% bovine serum albumin (BSA), 2% normal goat serum (NGS) in PBS for 30 minutes. Primary antibody IIH6 against α-DG in 1% BSA at 1:600 dilution was incubated 4 hours at RT or overnight at 4°C. Samples were washed three times for 10 minutes with PBS and finally incubated with Alexa Fluor 488-conjugated goat anti-mouse IgM or Alexa Fluor 594-conjugated goat anti-mouse IgM secondary antibodies (Life Technologies, Carlsbad, CA, USA) at 1:600 dilution. Samples stained without primary antibody were used as control.

### Western blot

The level of KRAS expression was assessed by subjecting 60 μg each of total cell lysates to immunoblot analysis. Cells were lysed in Triton lysis buffer containing 1% Triton X-100, 50 mM Tris pH 8, 150 mM NaCl, 1 mM EDTA, and 1x protease Inhibitor Cocktail (Sigma). After clarification of the lysates by centrifugation at 14,000 rpm for 10 min at 4°C, protein concentration of the lysates were measured using the Bradford method (Bio-Rad Laboratories). Samples were then electrophoretically separated on a 4–15% Criterion^™^ Tris-HCl 18-well gel, (3450028, Bio-Rad Laboratories) and transferred onto supported nitrocellulose membrane. Immunoblots were probed with anti-pan Ras C-4 primary antibody sc-166691 (Santa Cruz) or anti-KRAS primary antibody 12063-1-AP (Proteintech) at 1:1000 dilution in 3% nonfat dry milk PBS. Rabbit polyclonal antibody to actin AC-74 (Sigma) or anti-GAPDH PA1-988 (Thermo) was used at 1:3000 dilution in 5% nonfat dry milk/1xTBS-0.05% Tween. The blots were incubated with primary antibody overnight at 4°C. After wash, membranes were subsequently incubated with secondary antibodies of HRP-conjugated goat anti-mouse IgG (1:3000), or goat anti-rabbit IgG (1:3000) in their blocking buffer for 1 hour 30 min. Bands were detected using ECL detection Kit NEL 104001EA (PerkinElmer).

### Quantitative real time PCR assay

RNA was extracted from cells using TRIzol (Invitrogen) following the supplied protocol. Final RNA pellet was re-suspended in RNAse-nuclease free water. RNA concentration was determined using Nanodrop 2000c. 500 ng of RNA was subsequently converted to cDNA using the High-Capacity RNA-to-cDNA^™^ Kit (Applied Biosystems) following the supplied protocol. cDNA was then used for quantitative real-time PCR using the human Taqman assay for KRAS (Hs00364284_g1), with primer limited GAPDH (Hs02786624_g1) as the internal control and TaqMan^®^ Universal Master Mix II, with UNG (Life Technologies). Real time PCR was run on the BioRad CFX96 Touch^tm^ Real-Time PCR Detection System (BioRad) following the standard real time PCR conditions suggested for Taqman assays. Results of all expression levels were calculated as the 2^-ΔΔCt and compared to untreated control cells [26].

### Statistical analysis

All data are expressed as mean±SEM unless stated otherwise. Statistical analyses were performed with GraphPad Prism version 7.01 for Windows (GraphPad Software). Individual means were compared using unpaired t tests. Differences were considered to be statistically significant at p ≤ 0.05 (*).

## Results

### Ribitol enhanced matriglycan in breast cancer cell line is linked to cellular metabolism

**Fig 1:**
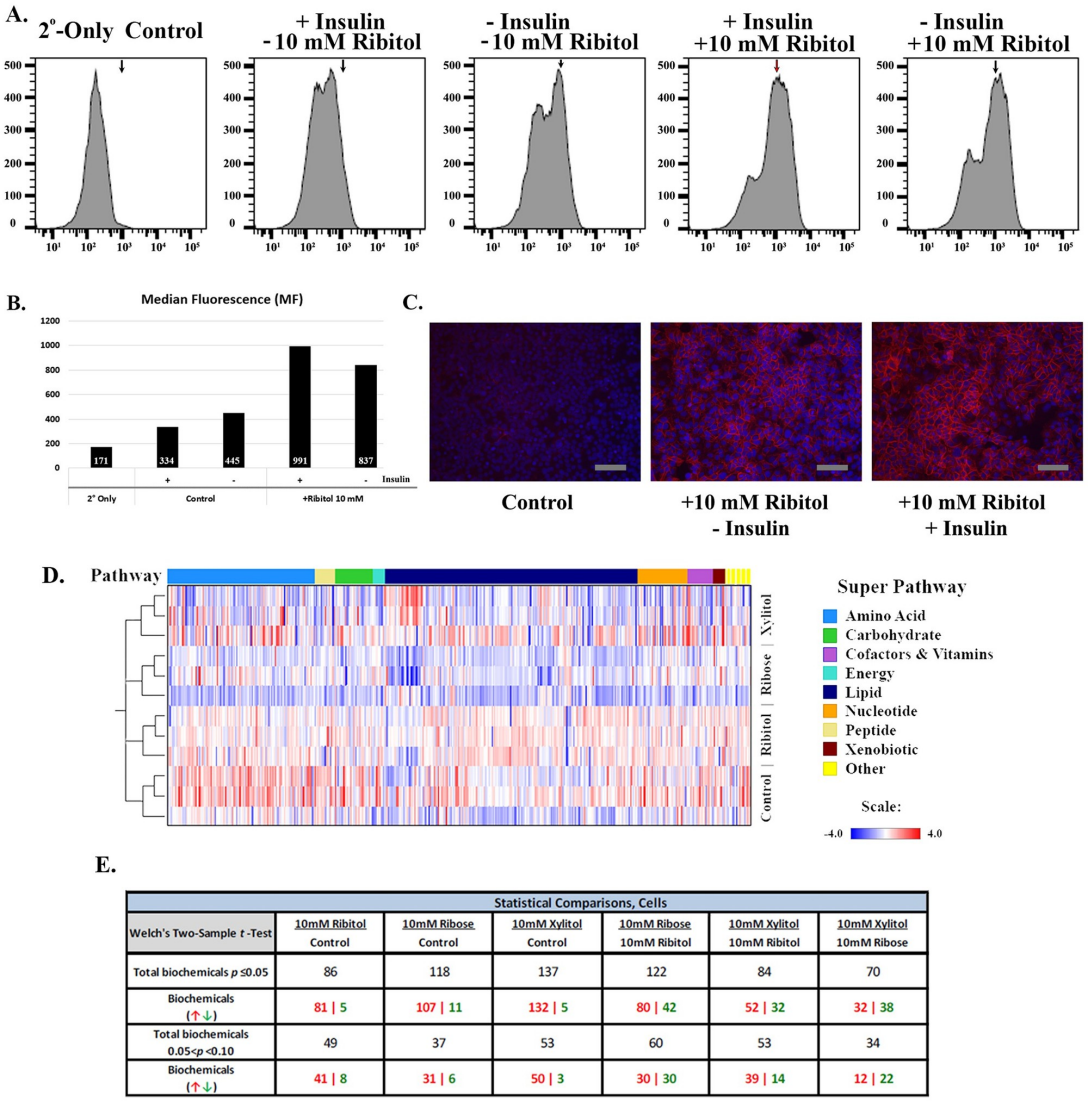
Matriglycan expression in MCF-7 cells treated with ribitol. MCF-7 breast cancer cells were incubated for 72 hours with ribitol (10 mM) supplementation, with and without insulin in the base media, and analyzed by flow cytometry for detection of glycosylated α-dystroglycan (A) Representative histograms demonstrating the distribution and median X axis, fluorescence intensity; Y axis, number of cells. Red arrow indicates peak of highest MF, and black arrow indicate corresponding position in each histogram. (B) Mean fluorescence intensity of MCF7 cells after ribitol treatment. (C) Representative micrographs of IIH6 ICC staining for glycosylated matriglycan in untreated control and 10 mM ribitol supplementation with and without insulin in culture media. 2^nd^ Ab-only control denotes staining with secondary antibody only, scale bar represents 200 μm. Metabolomic analysis of MCF7 cell lysates. (D) Hierarchical clustering analysis (HCA) heatmap of global metabolic profile alterations by pathway, amongst MCF-7 cells treated with 10 mM ribitol, 10 mM ribose, and 10 mM xylitol. (E) Comparison of statistically significant biochemical changes amongst metabolomic profiles of MCF-7 cells treated with ribitol, ribose, and xylitol. Cellular lysates compared by treatment to control, and between treatment groups. The two conditions compared make up the first row, with the total number of biochemicals reaching significance by two-sample t-Test p≤0.05 depicted in the second row, delineated by those increased or decreased in the third row. The count of biochemicals approaching significance, with two-sample t-Test 0.05<p<0.10 are depicted in the fourth row, and their change as increase or decrease separated in the fifth row.

We have demonstrated that supplement of ribitol results in enhanced matriglycan in breast cancer cells. Initial metabolic analysis of the treated MCF7 cells showed that exogenous ribitol is metabolized into ribitol-5P and CDP-ribitol, suggesting that ribitol likely participates in cellular metabolism. This raises a possibility that metabolic changes could also affect the expression of matriglycan on α-dystroglycan. We therefore performed a simple experiment to assess the effect of insulin in culture in the presence or absence of ribitol on matriglycan expression in MCF7 cells by flow cytometry. Insulin is known to stimulate cell cycle progression and DNA synthesis in MCF7 cells with enhanced utilization of glucose [27]. Removal of insulin from the culture media resulted in the cells having an enhanced expression of matriglycan with levels roughly 10% higher than the cells in the medium containing insulin (Fig 1A &1B). Interestingly, removal of insulin from the culture medium reduced response of the cells to 10 mM ribitol supplementation, with levels of matriglycan being about 10% lower than the medium containing insulin. (Fig 1B). This effect of insulin on α-dystroglycan expression was further confirmed by immunohistochemistry (Fig 1C). This data supports the hypothesis that alteration in metabolism itself affects expression of matriglycan, and ribitol effect can also be modulated by the metabolic status of breast cancer cells.

### Ribitol treatment modifies the metabolic profile of breast cancer cells

To assess the effect of ribitol on metabolism of cancer cells, we conducted a global untargeted metabolomic profile analysis by LC/MS/MS in breast cancer cell line MCF-7. The cell cultures were treated for 3 days with 10 mM ribitol, which was previously shown to induce high levels of matriglycan without toxicity [11]. Both cells and the culture media were harvested for measurement of metabolites. For comparison, effects of ribose and xylitol at the same concentration were also examined. As illustrated in Fig 1D, the number of metabolites with significant changes (p< 0.05) after ribitol treatment was 86 (19.8%) of assayed biochemicals with 94.2% of those being increased and 5.8% decreased compared to the controls. Significant changes were also detected with increasing numbers in ribose (118) and xylitol (137) treatments, with the majority also being increased in level (Fig 1E). Furthermore, treatment with the three metabolites produced unique patterns demonstrated by comparison between ribitol and the other two metabolites (Fig 1E). The most prominent difference was observed between ribitol and ribose treatments, with significant changes in 122 metabolites. While the changes in significant number of metabolites are expected with ribose and xylitol, a similar degree of alteration with ribitol was unexpected, but supports the hypothesis that ribitol actively participates in or alters cellular metabolism. More importantly the results demonstrate that each of these closely related pentose/pentitol has its own metabolic fingerprint in the cells. Several metabolic readouts, including cellular energetics, lipid metabolism and redox homeostasis will be discussed in more detail below.

### Ribitol enhances glycolysis with increase in pyruvate and lactate production: Effects on metabolites in glycolysis and pentose phosphate pathway (PPP)

**Fig 2:**
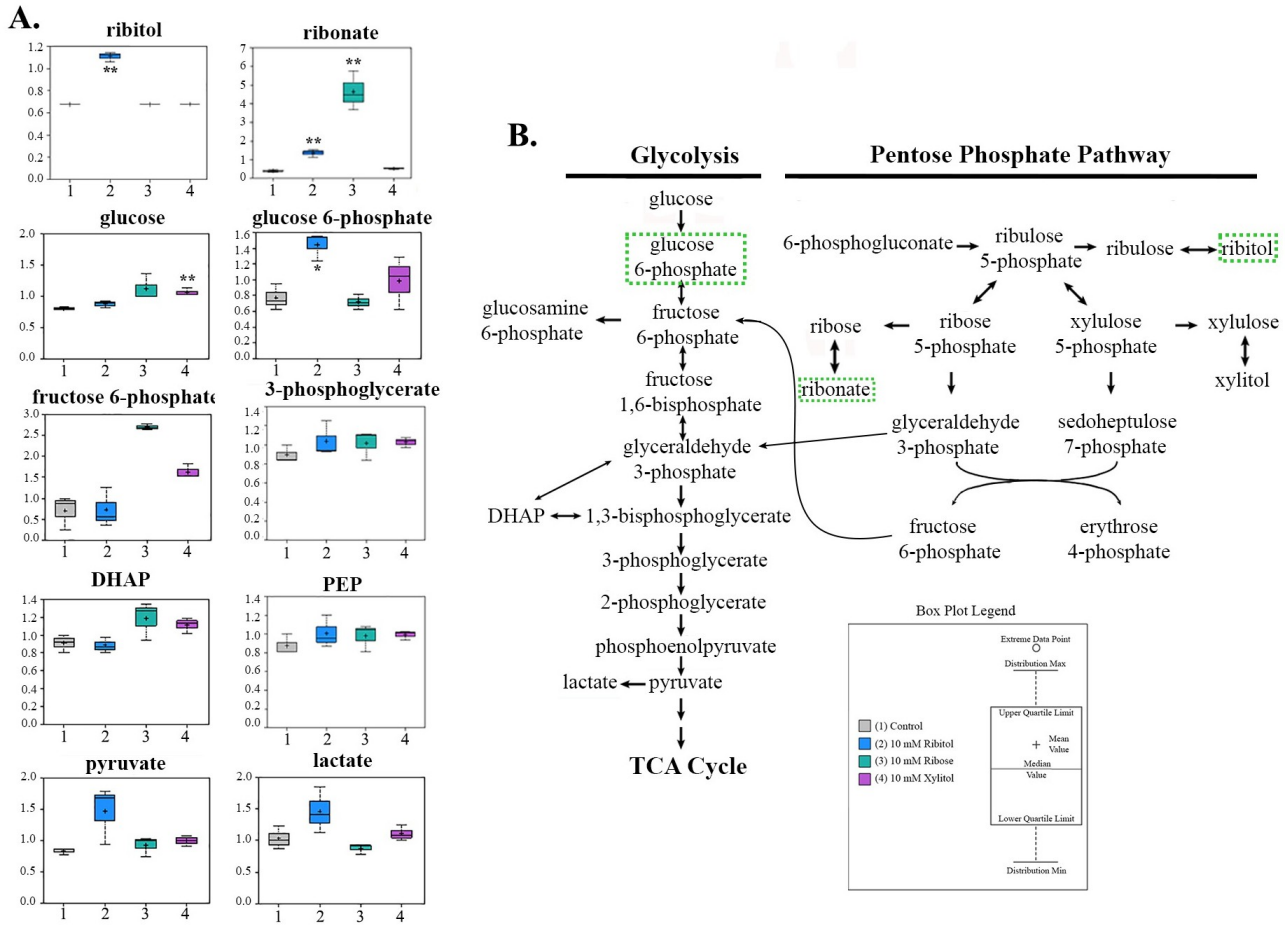
Alterations in glycolysis and the pentose phosphate pathway amongst metabolomic profiles of MCF-7 cells treated with ribitol, ribose, and xylitol. (A) Alterations of various glycolysis intermediates after MCF7 cells treated with ribitol, ribose and xylitol. (B) Hypothetical model of PPP and glycolysis metabolomic interactions after MCF7 cells were treated with ribitol. Significant *p>0*.*05* changes with ribitol indicated with increases in green in pathway schematic. Significance denoted by *p ≤ 0.*05*, **p ≤ 0.*01*, ***p ≤ 0.*001* within individual metabolite box plots, as determined by Welch’s two-sample t-Test.

As expected, ribitol treatment led to a significantly higher level of cellular ribitol when compared to control. Interestingly, one of the most significant changes observed was the level of glucose 6-phosphate (G6P), a metabolic intermediate shared by both PPP and glycolysis. Ribitol treatment resulted in 1.86 fold increase in levels of G6P (Fig 2A), but no change in fructose 6-phosphate (F6P) when compared to control. The down-stream glycolysis metabolites PEP and G3P were slightly elevated. In contrast, levels of pyruvate (1.76 fold) and lactate (1.42 fold) were significantly increased with ribitol treatment compared to the control (Fig 2A). Cellular glucose level was slightly increased in response to ribitol treatment. Interestingly, levels of glucose and pyruvate in the culture medium were lower in the presence of added ribitol when compared to the control (S1 Fig in S1 File). Therefore, exogenous ribitol enhances uptake of glucose and likely pyruvate as well, as both are abundant in the DMEM medium. Lactate levels also increased in the culture medium, further supporting enhanced glycolysis. However, intermediate metabolites in the oxidative branch of PPP down-stream of G6P were either not detected or without significant change. Metabolites in the non-oxidative branch of PPP including ribose, and arabitol/xylitol, were largely unchanged. Sedoheptulose 7-Phosphate (SH7P), an important convertible intermediate between PPP and glycolysis, was slightly lower in the ribitol treated cells than in the control cells. These data together suggest that the presence of excessive levels of ribitol and its derivatives may have limited effect on the downstream flux of the oxidative branch of PPP, but have major effect on the non-oxidative branch, contributing to the accumulation of G6P.

The unique effect of ribitol on both glycolysis and PPP was best illustrated when compared to the effect of ribose and xylitol treatments. In contrast to ribitol, the same concentration of ribose significantly increased the cellular levels of F6P, but not G6P. Furthermore, no increase was observed in the levels of pyruvate and lactate, although PEP and 3PG were also slightly elevated as observed with ribitol treatment. Cellular glucose levels were slightly higher in the cells with ribose treatment than the cells with ribitol treatment. There was no clear difference in levels of ribitol and arabitol/xylitol with ribose treatment when compared to ribitol treatment and the control. In contrast to ribitol treatment, SH7P level is slightly higher when compared to the control. Also interesting is that xylitol treatment elevated the levels of both F6P and G6P, but with no change in levels of pyruvate and lactate. These results clearly demonstrate unique effect of individual pentose and pentitol on these two metabolic pathways in the cancer cells.

### Ribitol dysregulates TCA with a decrease in citrate, but an increase in succinate and fumarate

**Fig 3:**
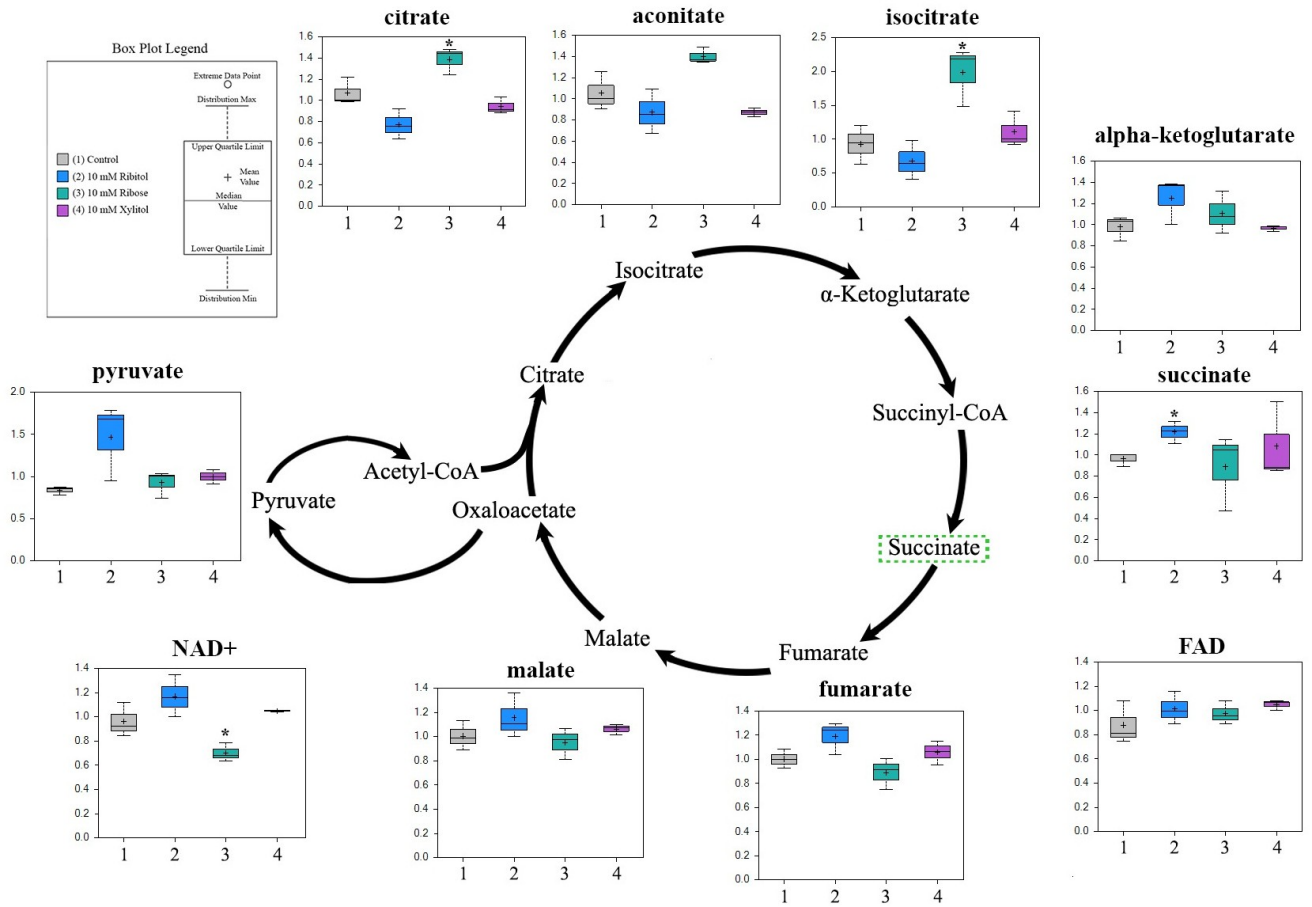
Alteration in TCA Cycle metabolite or intermediates in MCF-7 cells treated with ribitol, ribose, and xylitol. High level of pyruvate is associated with lower levers of citrate, isocitrate, indicating the conversion of pyruvate to acetyl-CoA is impeded. Increased level of alpha-KG to malate with ribitol treatment suggests an enhanced glutaminolysis and/or gluconeogenesis. Significant *p>0*.*05* changes with ribitol indicated with increases in green and decreases in red in pathway schematic. Significance denoted by *p ≤ 0.*05*, **p ≤ 0.*01*, ***p ≤ 0.*001* within individual metabolite box plots, as determined by Welch’s two-sample t-Test.

The most significant change in TCA metabolites with ribitol-treatment is the decrease in cellular levels of citrate, isocitrate and aconitate as compared to control (Fig 3). This reduction can either be the result of decreased levels of precursor Acetyl CoA converted from pyruvate, or increased down-stream consumption. However, Acetyl CoA was not identified, therefore, it cannot be assessed whether conversion from pyruvate to Acetyl CoA was enhanced or reduced, although failure to detect the metabolite itself indicates that the level was probably not elevated. Increased consumption of citrate is also unlikely with the fact that levels of most subclasses of fatty acid for which citrate is the major precursor were not increased or slightly reduced (Fig 3). The remainder of the detected TCA metabolites, from α-ketoglutarate, succinate, fumarate to malate were all elevated in the ribitol-treated cells. Conversely, ribose treatment enhanced activity of TCA cycle with increases of citrate, isocitrate and aconitate, but did not show apparent effect on the levels of other metabolites. The increase in citrate was well corelated with the significant increase in the levels of many fatty acid molecules (see description below). No significant alteration was observed in all detected metabolites by xylitol treatment as compared to control cells.

### Ribitol treatment enhances gluconeogenesis and nucleotide synthesis

**Fig 4:**
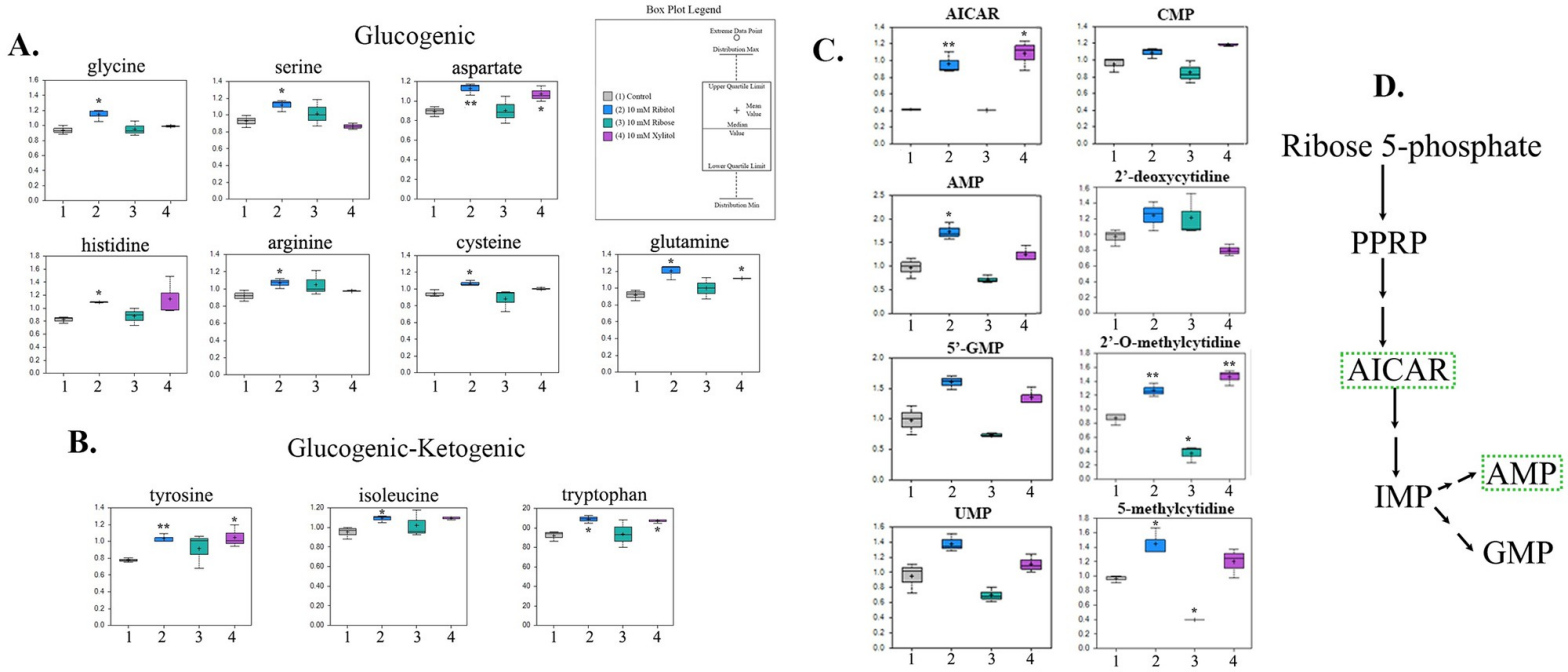
Alteration of glucogenic and glucogenic-ketogenic metabolites and nucleotide metabolism in MCF-7 breast cancer cells, treated with ribitol, ribose, and xylitol. (A) All glucogenic amino acids levels were increased after ribitol treatment compared to ribose and xylitol. (B) Glucogenic and ketogenic amino acids such as tyrosine, phenylalanine and tryptophan levels were increased in ribitol treated cells compared to ribose and xylitol treatment. (C) Ribitol treatment increases the nucleotide synthesis by increasing the purines and pyrimidines synthesis, compared to ribose and xylitol treatment and Ribitol treatment significantly increased the level of 5-aminoimidazole-4-carboxamide ribonucleotide (AICAR), one of the key intermediates of nucleotide synthesis. Significance denoted by *p ≤ 0.*05*, **p ≤ 0.*01*, ***p ≤ 0.*001* within individual metabolite box plots, as determined by Welch’s two-sample t-Test.

Cancer cells can generate energy (ATP) from alternative fuels other than glucose, a capacity crucial for their survival and proliferation. Given the fact that gluconeogenic and glycolytic pathways share common intermediates, gluconeogenesis might potentially be modified by ribitol treatment. Indeed, levels of all glucogenic amino acids as well as glucogenic and ketogenic amino acids were increased in MCF7 cells treated with ribitol when compared to control (Fig 4A). Particularly, the increase in glucogenic amino acids arginine (~1.2 fold), aspartate (~1.3 fold), cysteine (~1.1 fold), glutamine (~1.3 fold), glycine (~1.2 fold), histidine (~1.3 fold), and serine (~1.2 fold) was significant compared to control (Fig 4A). Also, glucogenic and ketogenic amino acids such as tryptophan (~1.2 fold), tyrosine (~1.3 fold) and isoleucine (~1.1 fold) increased significantly compared to control (Fig 4B). These amino acids have different entry points to the TCA cycle and can also be converted to different glycolytic intermediates for energy production.

Serine and glycine are biosynthetically connected and can be produced from the glycolytic intermediate 3PG which was also increased with ribitol treatment. Serine and glycine are primary sources of one-carbon units used in the synthesis of nucleic acids, and restoration of the NADPH/NADP^+^ ratio (Fig 4C). Consistently, ribitol treatment significantly increased the level of 5-aminoimidazole-4-carboxamide ribonucleotide (AICAR, ~2.3 fold), one of the key intermediates of nucleotide synthesis (Fig 4C). AICAR is a strong activator for AMP-activated protein kinase (AMPK), which is a potent energy sensor that promotes metabolic changes to ensure energy balance based on nutrient availability. Additionally, key metabolites of de novo synthesis of purines and pyrimidines such as Adenosine monophosphate (AMP) increased significantly (~1.8fold) in ribitol-treated cells compared to control. Guanosine monophosphate (GMP), dTMP (1.4 fold), orotate (1.8 fold) and CTP levels were all elevated with or without statistical significance (Fig 4C). Together, these data suggest that ribitol is able to remodel generation of energy and building blocks through a distinctive regulatory mechanism. These changes are consistent with the improved cell proliferation by ribitol.

In contrast to ribitol, ribose treatment was not associated with clear increase in the levels of glycine, serine, aspartate, cysteine, glutamine glycine, and histidine although the level of arginine was slightly higher than the control. Similarly, no increase in the levels of tryptophan, tyrosine, isoleucine and AICAR was detected with ribose treatment (Fig 4). Xylitol treatment increased the level of all aforementioned glucogenic amino acids, but at lower degree than ribitol treatment. However, levels of the glucogenic and ketogenic amino acids were similarly high in the cells with xylitol treatment as those with ribitol treatment, while no increase in AICAR and the nucleotides was observed.

### Ribose, but not ribitol increases fatty acid levels: Metabolites in lipids and phospholipid synthesis

The overwhelming majority of medium and long chain fatty acids including octanoylcamitine (C8), Azelate (C9:DC), myristate (14:0), Palmitate (16:0), margarate (17:0), stearate (18:0), nonadecanoate (19:0), arachidate (20:0), and EPA (20:5n3) in the cells with ribitol treatment remained at levels similar to that of the control (S2 Fig in S1 File). In contrast, significant increase in the levels of these fatty acids was detected in the cells treated with ribose. Similarly, the majority of phospholipids, including the subclasses of glycerophosphocholines (GPCs), glycerophosphoinositols (GPIs), glycerophosphoglycerol (GPGs), and glycerophosphoethanolamines (GPEs) were at similar or slightly elevated levels in the cells treated with ribitol when compared to the control, whereas the levels of these phospholipids were significantly higher in the cells with ribose treatment. However, there were a few exceptions. For example, levels of decosapentanoate (n6 DPA;22:5n6) were significantly higher in the cells treated with ribitol than that in the control and ribose-treated cells. The levels of esphingomyelins (SPHs) in the ribitol treated cells were also at the levels similar or slightly higher than that in cells treated with ribose, and significantly higher than the control. Consistent to the increased levels of serine with ribitol treatment, levels of glycerophosphoserines (GPS) were also slightly higher than the controls (S3 Fig in S1 File). Overall, the sharp contrast in levels of most lipids and phospholipids between the cells treated with ribitol and ribose is consistent with the decrease and increase in levels of citrate with the two compounds, respectively. Limited changes were observed in most of the detected lipids and phospholipids in cells treated with xylitol as compared to the control.

### Dysregulation in redox-related metabolites by ribitol

**Fig 5:**
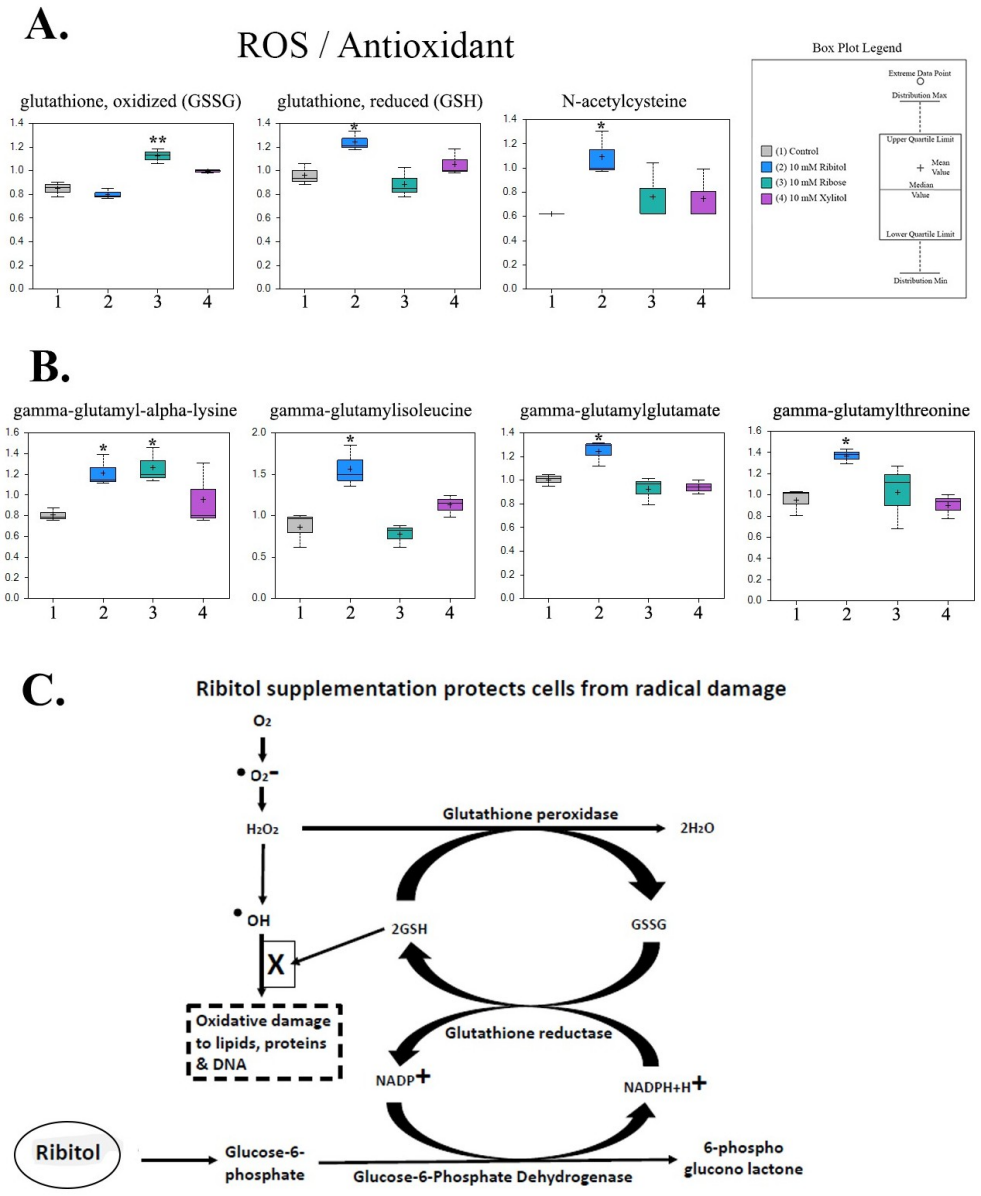
Alteration of glutathione metabolism in MCF-7 breast cancer cell line, treated with ribitol, ribose, and xylitol. (A) Ribitol treatment of MCF7 cells leads to increased levels of reduced glutathione (GSH) and decreasing the oxidized glutathione (GSSG), this is completely contrast to ribose treatment. (B) Box plot levels of gamma-glutamyllysine and gamma-glutamylisoleucine and gamma-glutamylglutamate, gamma-glutamylthreonine under ribitol, ribose, and xylitol treatments. (C) Hypothetical model of ribitol treatment increases glucose-6-phosphate which convert to 6-phosphogluconolactone by generating NADPH+H^+^. In the presence of glutathione reductase and NADPH+H^+^, oxidized glutathione is converted to reduced glutathione which prevents oxidative damage to lipids, proteins, and DNA. Significance denoted by *p ≤ 0.*05*, **p ≤ 0.*01*, ***p ≤ 0.*001* within individual metabolite box plots, as determined by Welch’s two-sample t-Test.

One important change with ribitol treatment is the alterations in Redox homeostasis. Ribitol treatment resulted in higher levels of reduced glutathione (GSH), and a lower level of oxidized glutathione (GSSG) when compared to the control, 1.29 p = 0.02 and -0.94 p = 0.34 respectively. (Fig 5A). GSH is one of the major cellular antioxidants and its increase could indicate upregulation of synthesis or recycling. Glutathione reductase can recycle GSSG to the reduced form with the concomitant oxidation of NADPH to NADP^+^. Because PPP is the main source for the production of NADPH, the main reducing agent for GSSG to GSH conversion, accumulation of GSH would suggest an increased flux of oxidative branch of PPP activated by the addition of ribitol. However, this hypothesis could not be directly supported as NADPH and NADP were not detected, and the other metabolites of the oxidative branch not identified in the study. Alternatively, increases in GSH could be the result of enhanced production. This is supported by the elevations in gamma-glutamylcysteine and other gamma-glutamyl amino acids (e.g. gamma-glutamyllysine and gamma-glutamylisoleucine and gamma-glutamylglutamate, gamma-glutamylthreonine) with ribitol treatment, (1.24–1.82 fold, p = 0.004–0.037) (Fig 5B). Another possibility is that an increased ratio of GSH/GSSG represents a decreased consumption of GSH in the ribitol treated cells due to reduced oxidative stress under a more glycolytic status as illustrated in Fig 5C. In contrast, ribose treatment increased levels of GSSG with slight decrease in GSH (see below), whereas xylitol treatment showed minimal effect on the levels of these metabolites. Overall, the GSH/GSSG profiles with ribitol and ribose treatment are consistent with the changes of metabolites in glycolysis and TCA pathways, specifically enhanced glycolysis with increase in levels of pyruvate, lactate and NAD+ with treatment of ribitol, but not ribose.

### Differential gene expression profiling of MCF7 cells treated with ribitol, ribose and xylitol

**Fig 6:**
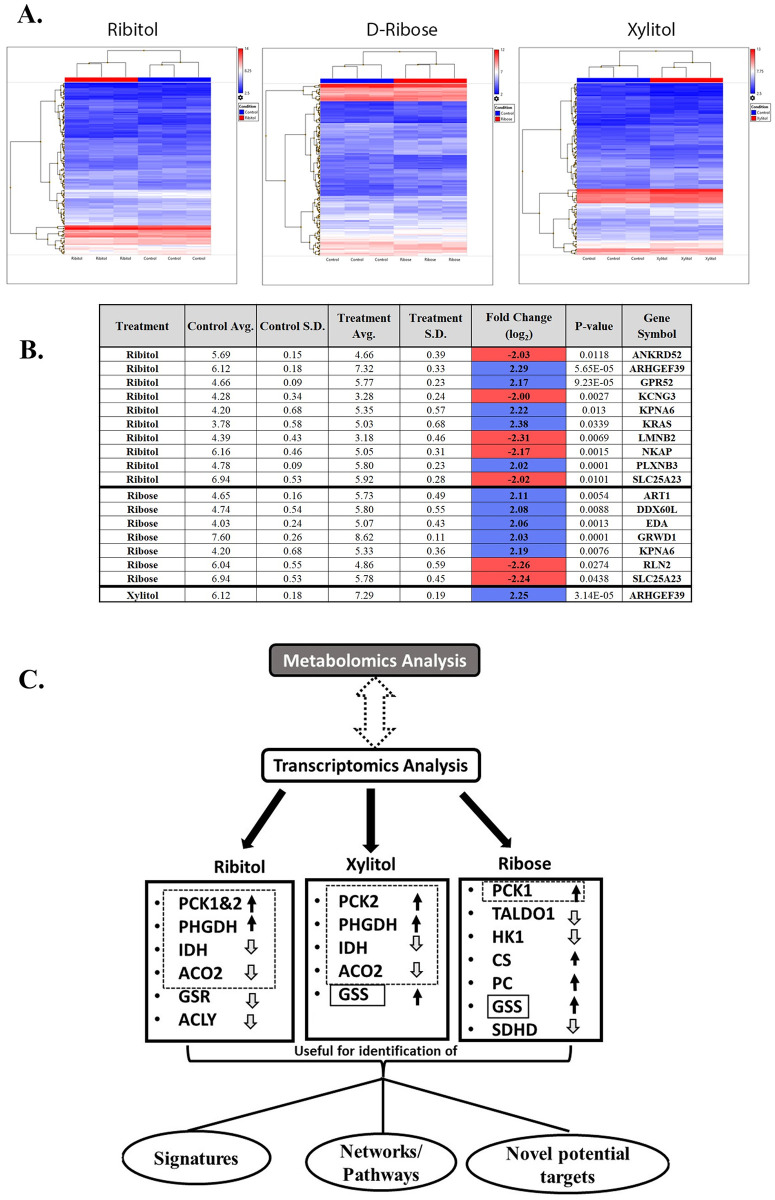
Heatmap of entire gene expression profiles of MCF-7 cells treated with ribitol, ribose, and xylitol by microarray analysis. (A) Biological replicates labeled by treatment group across the X-axis and significantly altered genes on the Y-axis. (B) Expression is depicted by intensity, while differential expression increased or decreased is depicted by color. (FC ≥2, significant *p>0*.*05* changes with ribitol indicated with increases in blue and decreases in red. Illustration of significantly altered transcripts by pentose sugars in breast cancer cells. (C) The genes listed represent the most significantly altered genes in each treatment when compared to the control and plays crucial roles in central carbon metabolism. Genes with alteration shared with ribitol, xylitol and ribose are indicated in dotted line squares and one gene shared between xylitol and ribose is indicated in solid line squares. The differential gene expression of these pentose sugars may be useful for identification of molecular signatures, analysis of networks or pathways and novel potential targets for treatment of cancer. Abbreviations: PCK1&2: Phosphoenol pyruvate carboxykinase 1&2; IDH: Isocitrate dehydrogenase; ACO2: Aconitase 2; GSR: Glutathione reductase; PHGDH: Phosphoglycerate dehydrogenase; ACLY: ATP citrate lyase. TALDO1: Trans aldolase 1; HK1: Hexokinase1; CS: Citrate synthase; PC: Pyruvate carboxylase; GSS: Glutathione synthetase; SDHD: Succinate dehydrogenase; LDH: Lactate dehydrogenase.

To assess whether the differential profiles in metabolites of MCF7 in response to the treatment of ribitol, ribose and xylitol involve changes in gene expression profile, RNA extracts from MCF-7 cultures treated with the same concentration of each compound (10 mM), and untreated control were subjected to Clariom S ^™^ transcriptome assay (Fig 6). Differential expression with significance was initially chosen using both criteria of fold change cutoff of +/- 2 and *P* ≤ *0*.*05*. Treatment with ribitol resulted in differential expression of 10 genes when compared to control. Of these, KRAS, ARHGEF39, KPNA6, GPR52, and PLXNB3 showed increased expression while KCNG3, ANKRD52, LMNB2 showed decreased expression (Fig 6A & 6B). Pathway analysis indicated that KRAS increases association with 51 processes, LMNB2 with 7, and PLXNB3 with 1 process. Of these processes, 37 had p-value ≤ 0.05. Three (3) pathways were identified with more than one gene involved, LMNB2 (down) and KRAS (up) being miR-targeted genes in epithelium, muscle cell, and lymphocytes. While the increased expression of KRAS, ARHGEF39, KPNA6 and decreased expression of NKAP are consistent with improved proliferation of the cells with ribitol treatment, none of the genes are directly related to the pathways to which significant changes in metabolites were detected with ribitol treatment.

Treatment with Ribose resulted in differential expression of 7 genes when compared with control (Fig 6B). Of these, KPNA6, ART1, DDX60L, EDA, and GRWD1 showed increased expression, while SLC25A23, and RLN2 showed decreased expression. Pathway analysis indicated a total of 7 pathways, 6 of which had p-value of ≤ 0.05. Of these, EDA expression was related to 6 pathways with the most significant effect on signaling in hair follicle development, induction, and organogenesis. ART1 upregulation was associated with Defensins pathways, whereas the effect on other pathways was not significant. Again, genes directly involved in PPP, glycolysis and TCA were not identified as differentially expressed between ribose treatment and control. Treatment with xylitol resulted in differential expression in a single gene ARHGEF39 with upregulation as compared to control and involvement of specific pathway was not indicated.

Comparing the genes differentially expressed with significance between the treatments, only 3 of them were shared by two or more treatment. SLC25A23 was down-regulated and KPNA6 up-regulated to a similar degree by both ribitol and ribose, whereas ARHGEF39 was upregulated by ribitol and xylitol. There was no similarity between xylitol and ribose treatments (Fig 6A and 6B). The limited similarity in gene expression profile between each metabolite treatment is therefore consistent with distinctive patterns in alteration in metabolite profiles of each compound.

### Transcriptome profiles of genes regulating glycolysis, PPP, TCA and glutathione

To further assess whether the remodeling of metabolites with ribitol supplement is linked to changes in gene expression and considering that limited change in level of enzymes could well be significant for levels of its substrates, we analyzed 240 genes functioning as enzymes in the metabolic pathways applying single *P<0*.*1 and P<0*.*05* as cutoff for significance without limit of fold change. We identified 11 genes with *P<0*.*05* and another 15 genes with *P<0*.*1 - >0*.*05* when comparing the cells treated with ribitol to the control (Fig 6C). Ribose treatment resulted in the greatest number of altered genes (18.3%, 44/240) whereas Xylitol treatment the least number of altered genes (10%, 24/240). These genes fell into those regulating glycolysis, gluconeogenesis, glutaminolysis, TCA pathways as well as GSH/GSSG levels (S1 Table in S1 File).

#### Glycolysis and PPP

Hexokinase 3 (HK3), a member of the sugar kinase family was significantly increased with ribitol treatment (Supplementary Material). HK3 catalyzes the rate-limiting and first obligatory step of glucose metabolism, ATP-dependently phosphorylate glucose to G6P. The significant increase was not observed with ribose, and in contrast, a decrease was detected with xylitol. The result therefore is in agreement with the level of G6P in the cells treated with the three different sugars. Phosphofructokinase (PFK) was up 1.13 fold (*P = 0*.*074*), catalyzing the phosphorylation of fructose-6-phosphate to fructose-1,6-bisphosphate, a key regulatory step in the glycolytic pathway and considered a high energy intermediate of the glycolysis pathway. Phosphoenolpyruvate carboxykinase 2 (PCK2) was up 1.32 fold (*P = 0*.*015*). The mitochondrial enzyme PCK2 catalyzes the GTP-driven conversion of OAA to PEP as a rate-limiting step regulating glycolysis, gluconeogenesis and TCA. PCK1 was also increased although with lower P value (*P = 0*.*065*). This upregulation could explain the increases in the level of PEP and decreased levels of citrate, and significant elevation of pyruvate. Since nearly all of the glycolytic reactions upstream of PEP and downstream of glucose-6-phosphate (G6P) are reversible, increase in PEP could effectively increase the levels of those intermediates, further fueling multiple biosynthetic processes including serine synthesis and nucleotide synthesis. Notably, PCK2 by promoting preferential conversion of lactate to OAA and further to PEP, increases energy production without increasing the flux of TCA even under low-glucose conditions. As a result, PCK2 activity could contribute to cell survival and growth by reducing oxidative stress. Phosphoglycerate dehydrogenase (PHGDH) was up 1.59 fold (P = 0.056) (S1 Table in S1 File). This enzyme catalyzes the oxidation of 3-PG from glycolysis to 3-phosphohydroxypyruvate (3-PHP), the first and only rate-limiting enzyme in the *de novo* serine biosynthetic pathway and for the synthesis of downstream glycine and cysteine. The significant upregulation of this gene therefore is highly consistent with the increased level of serine, glycine and cysteine in the ribitol-treated cells. The alteration in the expression of these genes overall is in agreement with the enhanced glycolysis in the cancer cells treated with ribitol (Fig 6C). In contrast, these 3 genes were all down-regulated more than 1-fold with ribose treatment. These genes were similarly upregulated with xylitol treatment as ribitol treatment (Supplementary data). Interestingly, 6-phosphofructo-2-kinase/fructose-2,6-biphosphatase 1 was upregulated (P = 0.06) with ribitol treatment, but not significantly with the other treatments, further supporting the enhancement of glycolysis by ribitol and oxidative phosphorylation by ribose.

#### TCA

Significant changes were also detected for genes involved in the TCA cycle. Aconitase 2 (ACO2), a mitochondrial protein was downregulated by 1.27 fold (P = 0.023). This protein catalyzes the interconversion of citrate to isocitrate via cis-aconitate in the second step of the TCA cycle. The reduction in expression of this gene is therefore consistent with the reduced levels of isocitrate and aconitate. ACO2 expression is reduced in breast cancer, and increasing the levels of the enzyme in MCF-7 cells can inhibit cell proliferation. Isocitrate dehydrogenase 3 (IDH3) was also downregulated by 1.18 fold (S1 Table in S1 File). IDH3 is NAD-dependent and catalyzes the irreversible conversion of isocitrate to α-KG while reducing NAD+ to NADH [13]. This down regulation would be against the notion that the increased availability of α-KG is largely coming from upstream isocitrate, but favors a source of glutaminolysis. The down regulation of these two genes probably reflects the significant reduction of the upstream citrate. This is supported by the significant down-regulation of ATP citrate lyase. Consistently glutamate pyruvate transaminase (GPT) was upregulated by 1.42 fold. This protein catalyzes the transfer of an amino group from L-alanine to α-ketoglutarate, the products of thisreversible transamination reaction being pyruvate and L-glutamate. This again support that the cells utilize shunt pathways for the production of energy. Upregulation of this gene may also play an important role in amino acid metabolism and gluconeogenesis. Another enzyme of the TCA cycle, malate dehydrogenase 1B (MDH1B) was also upregulated 1.23 fold. MDH1B catalyzes the reversible oxidation of malate to oxaloacetate, utilizing the NAD/NADH cofactor system in the citric acid cycle. The significance of this upregulation to the levels of malate to oxaloacetate is not clear as the level of oxaloacetate was not determined. Both ACO2 and IDH3 were in contrast upregulated in the cells with ribose treatment, but down-regulated although with lower fold change in xylitol-treated cells.

#### Glutathione

There was a significant reduction in the levels of Glutamate Cysteine Ligase (GCL, -1.28 fold) and *Glutathione reductase* (*GR*. *-1*.*15 fold*). GCL catalyzes the first and rate-limiting step in the production of the cellular antioxidant glutathione (GSH) and *GR* reduces GSSG into GSH in the presence of NADPH and flavine adenine dinucleotide (FAD). The reduction of these two genes appears to be inconsistent with the enhanced levels of GSH in the ribitol treated cells. However, this would be consistent with our hypothesis that reduction in the levels of GSH does not result from the enhanced production, rather, this was a result of reduced oxidative stress with enhanced glycolysis and glutaminolysis. Another significant change with ribitol treatment was glutathione-specific gamma-glutamylcyclotransferase 1 (CHAC1) upregulation, a phosphoglycerate dehydrogenase (S1 Table in S1 File). CHAC1 catalyzes the cleavage of glutathione into 5-oxo-L-proline and a Cys-Gly dipeptide, thus reducing levels of glutathione. Glutathione depletion is important for apoptosis initiation and execution. This again would appear inconsistent with the increase in GSH level with ribitol treatment. However, this might well be a response to the increase of GSH, protecting cells from overly high levels of GSH. Alternatively, CHAC1 upregulation indicates possible ER stress under the presence of ribitol. In contrast, downregulation of GCL and GR was not significant with both ribose and xylitol treatment, and CHAC1 was downregulated with ribose treatment, supporting our hypothesis.

Significant change in transcript levels was also detected for glutamate receptor (GRM5) and EYA transcriptional coactivator and phosphatase 1 (EYA1), TDP-glucose 4,6-dehydratase (TDGS), and hydroxyacylglutathione hydrolase (HAGH) when compared the ribitol treated cells with the control, but their potential effect to the metabolic profile of the cells is not clear. The significantly greater number of genes altered with ribose treatment is consistent with its known effects on multiple metabolic pathways with promiscuity. Conversely, utilization of xylitol by the cells in culture is limited. Remarkably ribitol shared only 9 genes with ribose and 10 genes with xylitol treatments, indicating a diverse effect of each sugar on metabolism.

### KRAS upregulation with ribitol treatment and potential significance in breast cancer cells

Of the differentially expressed genes from the transcriptome results with ribitol treatment, KRAS upregulation stood out both for its role in cancer and cell biology, as well as its potential significance in response to ribitol supplementation (Fig 6B). We therefore further analyzed expression status of genes known to related to RAS related cell proliferation and growth inhibition using P value of <0.05 as single determinant. There were 15 genes with significant changes in the cells treated with ribitol. These genes can be divided into two groups, one with oncogenic or cell proliferation potential and the other considered as tumor suppressor. Interestingly, genes with oncogenic potential were either upregulated (AKTIP, 1.36 fold; HIF3A, 1.37 fold; PIKFYVE, 1.13 fold), or downregulated (PIK3CB, -1.21 fold; GRB2, -1.21 fold; MAP2K1, -1.13 fold) in the cells with ribitol treatment. Similarly, the identified tumor suppressors were also either up-regulated (TP53TG3B; TP53TG3C, 1.14 fold) or down-regulated (RASA3, -1.33 fold; RASSF2, -1.24 fold) (S2 Table in S1 File). It is therefore probable that alteration in expression of these genes reflects slightly improved cell growth by ribitol, rather than an oncogenic potential. Our prior study of ribitol treatment in dystrophic mice with maximal dose of 10g/kg daily for up to 1 year did not show any increased incidence of tumors in any tissue also suggesting that this effect might be specific to established cancer cells which are of unlimited growth potential [23].

**Fig 7:**
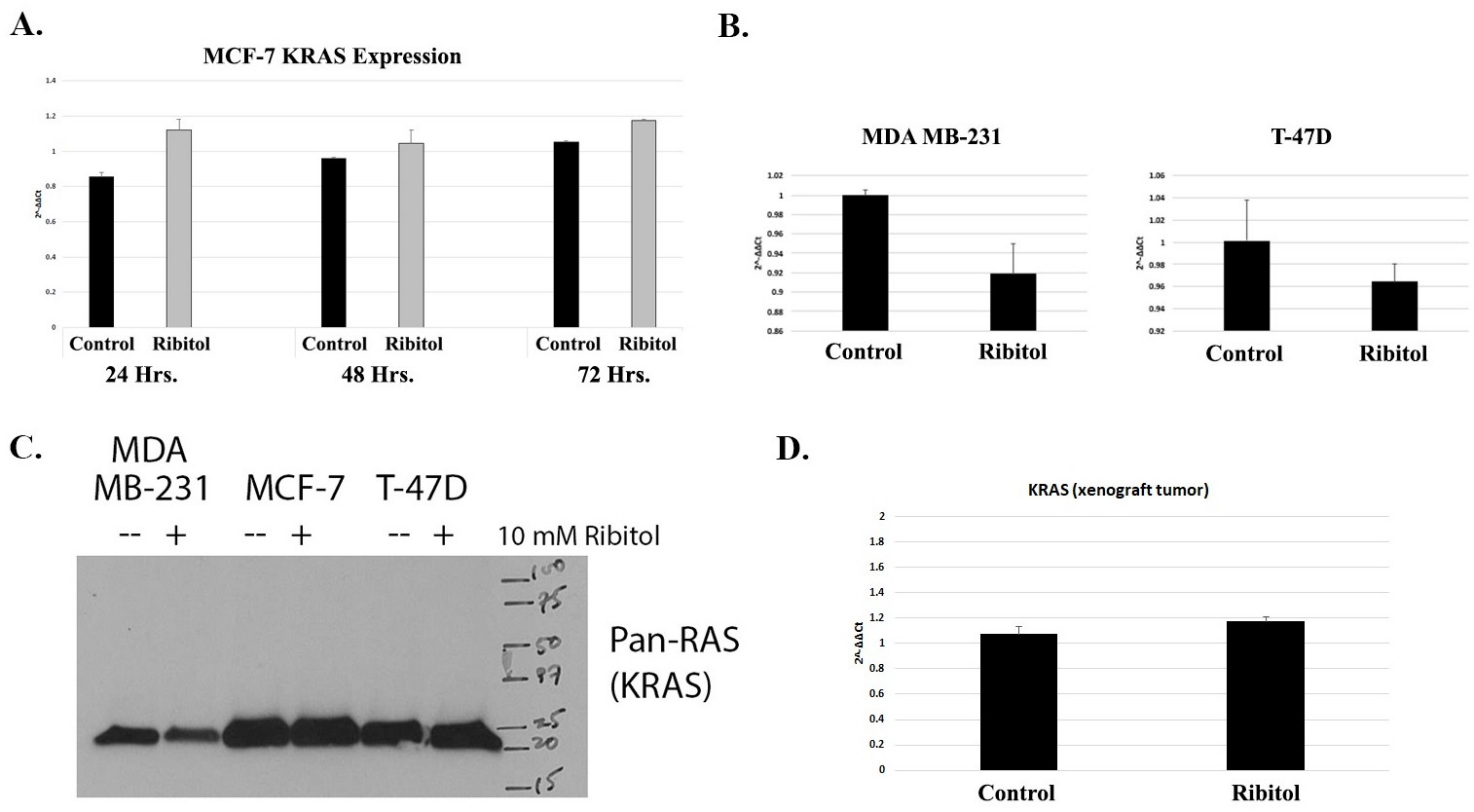
KRAS gene expression and protein levels in MCF-7, MDA MB-231, and T-47D breast cancer cells treated with ribitol. A) KRAS expression in MCF-7 cell line by Real-Time qRT-PCR B) Relative KRAS expression in MDA MB-231 and T-47D breast cancer cell lines by Real-Time qRT-PCR C) KRAS protein expression by western blot of control, and ribitol-treated breast cancer cell lines MDA MB-231, MCF-7, and T-47D (D) Relative KRAS expression in xenograft MCF-7 tumors after ribitol treatment by Real-Time qRT-PCR.

We confirmed that KRAS transcript expression is increased by ribitol treatment, by 24 hours after treatment and is continued through 72 hours, as quantified by Real Time-PCR (Fig 7A). Interestingly, when we assayed two additional breast cancer cell lines, MDA MB-231 and T-47D, we found that KRAS transcript levels were decreased (Fig 7B). We then measured the level of KRAS protein in the cells treated with ribitol by western blots and found that the levels of KRAS were not markedly different between the cells treated with ribitol and the control, suggesting that transcript levels may not be directly translated into protein levels, or that western blot is not sensitive enough to quantify these changes. In our previous study we examined the effects of ribitol on MCF-7 tumor cells in vivo through a xenograft study, to observe effects related to α-DG glycosylation.[11] The effect of ribitol on KRAS expression was also evaluated by Real-Time PCR on xenograft MCF-7 tumors in control mice and mice treated with 10% ribitol ad libitum for 45 days. Similarly to the cultured cells, slightly elevated KRAS expression was observed in the tumors treated with ribitol over untreated control (Fig 7D).

Ribose and xylitol treatment also showed altered oncogenesis-related genes, 37 and 24 respectively. Since these two sugars have long been used widely in human with no clear evidence of oncogenic consequence, no further discussion will be made. Interested readers are referred to the Supplementary data.

## Discussion

The role ribitol plays in metabolic pathways is largely unknown. Some species of bacteria can effectively convert ribitol and xylitol to ribulose and xylulose and then further phosphorylate to participate in major carbohydrate pathways such as glycolysis and TCA [28]. However, ribitol, although widely detected in tissues and fluids, is generally considered an end-product of metabolism in mammalian cells as limited studies have shown that ribitol cannot be converted to pentoses or other pentitols in cultured human cells although pentoses such as ribose can be converted into ribitol [29]. The current study showed almost no changes in levels of either ribitol or ribose when the cells were treated with either ribose or ribitol, respectively. Furthermore, substantial increase of arabinate and xylulonate was only detected in the ribose treated cells, with increased ribonate in cells treated with ribose and ribitol. The results therefore support the notion that ribitol participates in cellular metabolic pathways, but with limited capacity for free conversion into other known pentoses and pentitols. This is in contrast to ribose which is known to be readily convertible to other metabolites in several pathways [30].

Treatment failure in all breast cancer types occurs as a consequence of inherent or acquired resistance [31–34]. One of the paths to cancer cells becoming resistant to treatments is by rewiring their metabolism, to adapt to and survive in sub-optimal cellular and ECM environments imposed by therapeutic agents. Enhanced glycolysis even under normoxia or hyperoxia conditions (Warburg effect) is one of the major features of cancer cells to deploy for their survival and growth advantage over normal cells. Recent progress in understanding the mechanism involved in the Warburg effect has led to the proposal that alterations in metabolic pathways can potentially be exploited for therapeutic intervention to cancers. In this context, a better understanding of the process of cancer adaptations to individual drug treatment, which render cancer cells dependent on distinctive metabolic pathways, is clearly needed. This need can now be better served by combined analysis of transcriptomics and metabolomics, an emerging and fast evolving field in cancer study with the potential to better delineate metabolic pathways specific to cancer cells and in response to treatments. While ribitol appears to have limited effect on levels of most detected intermediates of PPP, ribitol treatment affects wide range of metabolic pathways from glycolysis, TCA, and redox balance, lipid and amino acid synthesis. Specifically, ribitol supplementation enhances glycolysis and partially inhibits oxidative phosphorylation flux with increased levels of GSH. These results for the first time provide clear evidence that ribitol can alter a wide range of metabolic pathways, resulting in a unique signature different from the closely related pentose and pentitol.

**Fig 8:**
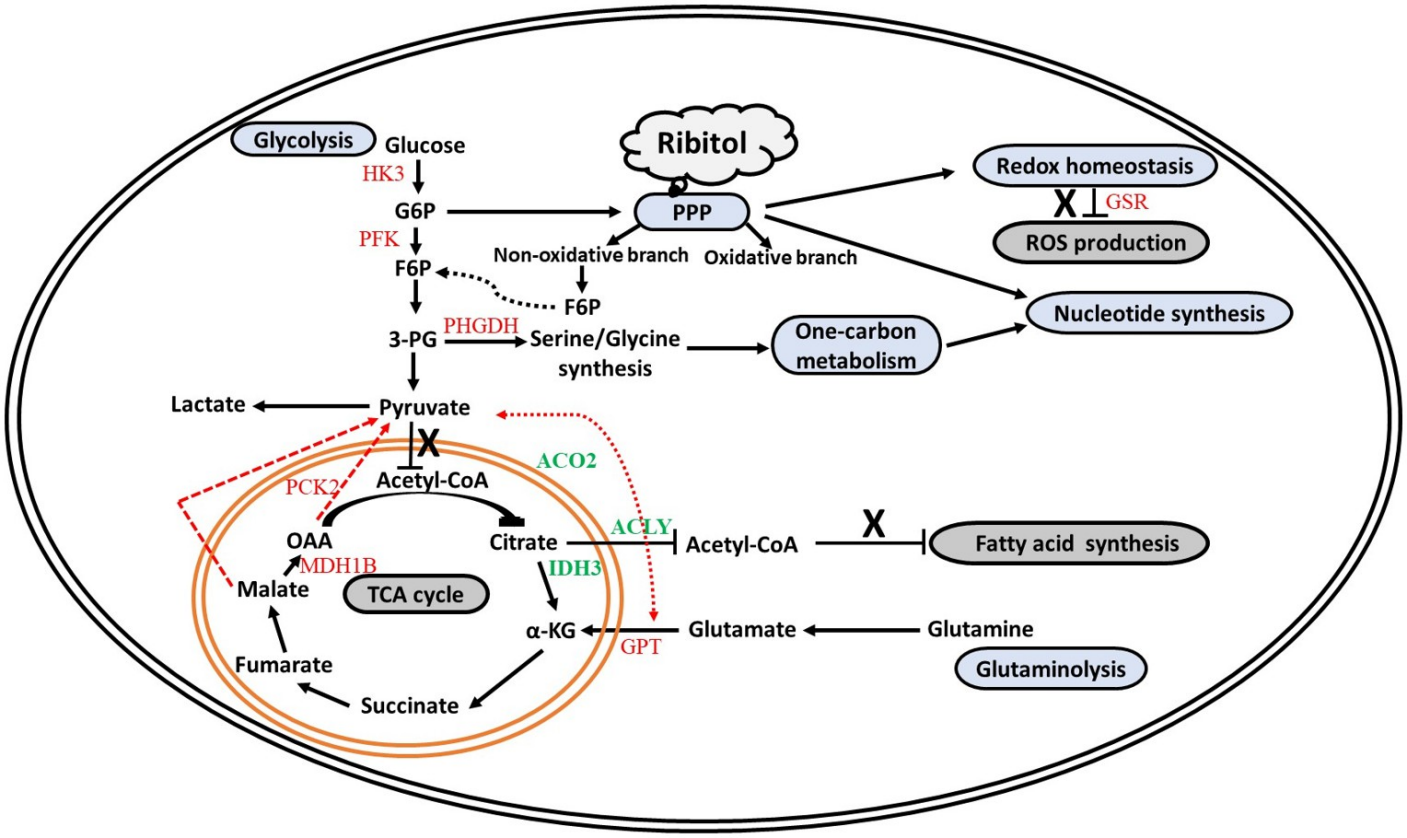
Schematic summary of metabolic and transcriptome alterations in MCF-7 breast cancer cells treated with ribitol. Ribitol treated MCF 7 cells enhances various central carbon metabolic pathways like glycolysis, one carbon metabolism and nucleotide synthesis. Simultaneously, ribitol treatment decreases the ROS production, not entering into the TCA cycle (Acetyl Co-A not assayed in our study) and no fatty acid synthesis further. In addition, some of the TCA cycle intermediates were altered, this could be due to increased glutaminolysis. This may fuel the TCA cycle intermediates and their levels were altered in ribitol treatment. Metabolic pathways and the observed alterations in metabolites are depicted in black, while the associated enzymes found to be altered via transcriptomics are depicted as decreased expression (green) and increased expression (red) upon ribitol administration.

One prominent signature of ribitol treatment in the breast cancer cells, and in contrast to ribose and xylitol treatment, is the significant increase in glucose-6-phosphate, a key intermediate shared by PPP, glycolysis and gluconeogenesis. This increase is associated with higher levels of downstream metabolites of glycolysis including pyruvate and lactate, indicating enhanced glycolysis. Other factors could also contribute to the increased levels of G6P including enhanced uptake of glucose as indicated by the lower levels of glucose in the culture medium with ribitol treated cells. This would be in agreement with a higher capacity of cancer cells to take up glucose than normal tissue to maintain the less efficient glycolysis as an energy source even in the presence of oxygen (aerobic glycolysis) [26]. The availability of abundant supplies of pyruvate in the medium could also retard the flux of glycolysis by a feedback mechanism, leading to accumulation of G6P (Fig 8).

To our surprise, increased pyruvate levels through enhancement of glycolysis in ribitol-treated cells is associated with reduced, rather than increased levels of intermediate metabolites of the TCA cycle, namely citrate, isocitrate and aconitate. In agreement, reduced rather than increased levels of fatty acids were detected in ribitol-treated cells. This is further supported by transcriptomic data showing a downregulated expression of ATP citrate lyase which is a key enzyme of de novo fatty acid synthesis. However, the levels of other metabolites of the TCA cycle from alpha-ketoglutarate to malate were all clearly increased. We hypothesize that this is likely the result of enhanced gluconeogenesis, glutaminolysis and use of ketogenic amino acids in ribitol treated cells. This is supported by significant increase of the common intermediates shared by gluconeogenic and glycolytic pathways including arginine, aspartate, cysteine, glutamine, and glycine. The overall effect and possible mechanisms of ribitol treatment on glycolysis and TCA are depicted in Fig 8. Such pathway alterations are unique to ribitol treatment, in contrast to ribose and xylitol treatment.

Another interesting finding of our study is that ribitol treatment, again in contrast to ribose treatment, significantly increases levels of reduced glutathione (GSH). This result is in agreement with an early report by Stone et al in 2014 [35]. The previous study aimed to assess the roles of polyols, specifically arabitol and ribitol, in the pathophysiology of a disease caused by ribose-5-phosphate isomerase deficiency [36]. In the investigation of oxidative homeostasis of the prefrontal cortex of rats, Stone et al reported that arabitol and ribitol increase activity of several antioxidant enzymes, including superoxide dismutase, catalase, and glutathione peroxidase. This together with our transcriptomic data showing downregulation in the expression of glutathione reductase and glutamate cysteine ligase (GCL) suggest that ribitol might be able to enhance antioxidant capacity in wider cell populations. However, the mechanism(s) of action is not entirely understood. Based on the data available, one conceivable path leading to the increase in GSH or antioxidant enzymes is illustrated in Fig 5. Ribitol enhances the uptake of glucose with increase in G6P which may consequently enhance the production of NADPH through the oxidative branch of PPP. Alternately, enhanced glutaminolysis can also increase the level of NADPH. Enhanced NADPH level in turn increases the level of GSH. At the same time, increased reliance of energy production on glycolysis and less on oxidative phosphorylation could help the cells maintain relatively higher reserves of the antioxidant GSH. The overall oxidative homeostasis with ribitol treatment therefore could have a profound effect on survival and health to both normal and cancer cells. Concurrently, an altered metabolomic status could provide a new venue for development of targeted therapeutic approaches in combination with other available targeted therapies. Furthermore, the overall effect of ribitol administration on cancer growth and maintenance may well depend on many other alterations ribitol might cause. An example is that ribitol treatment leads to significant accumulation of AICAR, a potent activator of AMP-activated protein kinase (AMPK). Enhanced expression of AICAR has been reported with anticancer properties including induction of apoptosis and inhibition of migration and invasion in prostate cancer cells [37]. It has also been previously demonstrated that AMPK activation by AICAR in MCF7 cells causes metabolic rearrangements, leading to antiproliferative and anticancer effects [38, 39]. Our long-term and high dose ribitol treatment in a mouse model of muscular dystrophy also shows no sign of increase in tumorigenesis in any tissue. Clearly long-term benefit or potential side effect of ribitol treatment needs to be further investigated.

This study investigated biochemical effects of a largely unknown metabolite ribitol in breast cancer cells with a combination of metabolomic and transcriptomic analyses. Ribitol, in contrast to ribose, specifically enhances glycolysis, dysregulates TCA cycle and increases level of antioxidants. The unique metabolic signature provides mechanistic insight and reprogramming specificity together with ribitol’s capability to enhance glycosylation of α-DG, opening new avenues for its potential toward cancer therapy and other applications. The limitation of the study is that the characteristics may well be defined by cellular types and culture environment.

## Supporting information

S1 File (PDF)

S1 Data (XLSX)

S2 Data (XLSX)
