# Supplementary material for: Ribitol alters multiple metabolic pathways of central carbon metabolism with enhanced glycolysis: A metabolomics and transcriptomics profiling of breast cancer
Source: PLoS One. 2022 Dec 7;17(12):e0278711. doi: 10.1371/journal.pone.0278711 (PMC9728907; doi:10.1371/journal.pone.0278711)
Supplement: S1 File — (PDF) [file pone.0278711.s001.pdf]

Supplementary Information

Ribitol alters multiple metabolic pathways of central carbon metabolism with enhanced glycolysis: A metabolomics and transcriptomics profiling of breast cancer

Tucker, Jason D[1], Doddapaneni, Ravi[1], Lu, Pei J[1]., Lu, Qi L[1*].

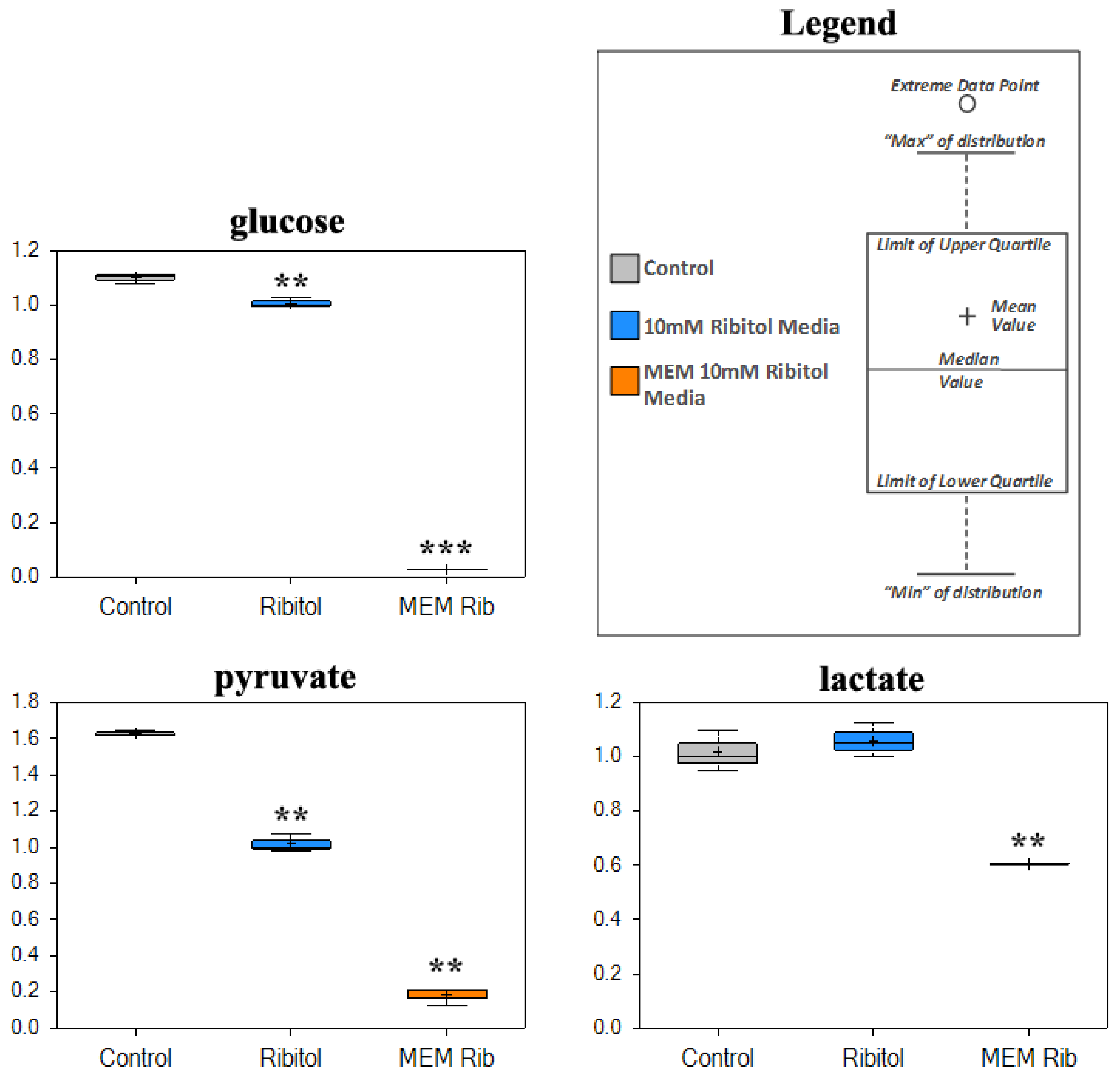

SI Figure 1: Alteration of metabolites associated with glycolysis in cell culture media with ribitol supplementation. Levels of glucose and pyruvate in the culture medium were lower in the presence of added ribitol in DMEM and MEM when compared to the control. Exogenous ribitol enhances cellular uptake of glucose and probably pyruvate as well, while lactate levels also increased in the culture medium, supporting enhanced glycolysis. Significance denoted by $*p \leq 0.05$, $**p \leq 0.01$, $***p \leq 0.001$ within individual metabolite box plots, as determined by Welch's two-sample t-Test.

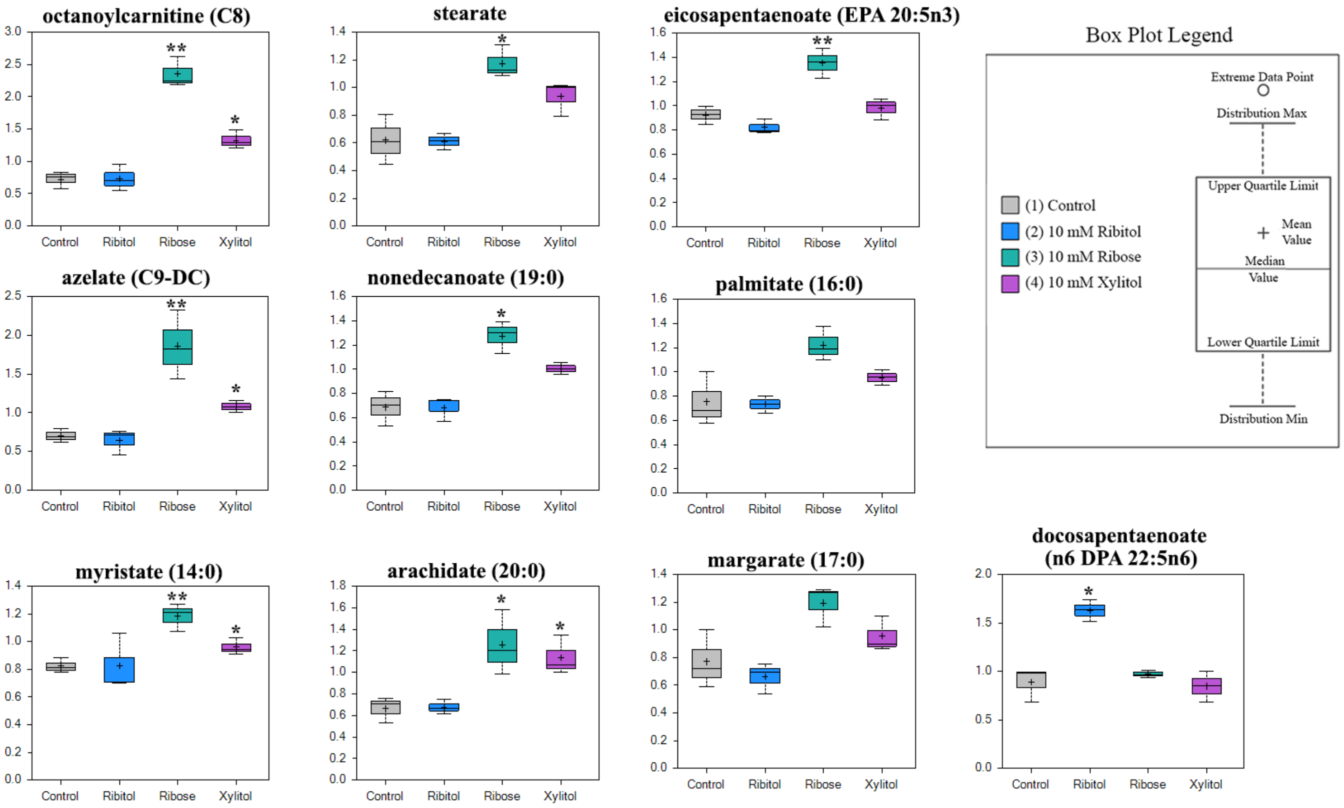

SI Figure 2: Ribose, but not ribitol increases fatty acid levels: metabolites in lipids and phospholipid synthesis

The overwhelming majority of medium and long chain fatty acids including octanoylcarnitine (C8), Azelate (C9:DC), myristate (14:0), Palmitate (16:0), margarate (17:0), stearate (18:0), nonadecanoate (19:0), arachidate (20:0), and EPA (20:5n3) in the cells with ribitol treatment remained at the levels similar to that of the control In contrast, significant increase in the levels of these fatty acids was detected in the cells treated with ribose. Similarly, most phospholipids, including the subclasses of glycerophosphocholines (GPCs), glycerophosphoinositols (GPIs), glycerophosphoglycerol (GPGs), and glycerophosphoethanolamines (GPEs) were at similar or slightly higher levels in the cells treated with ribitol when compared to the control, whereas the levels of these phospholipids are significantly higher in the cells with ribose treatment. Significance denoted by $*p \leq 0.05$, $**p \leq 0.01$, $***p \leq 0.001$ within individual metabolite box plots, as determined by Welch's two-sample t-Test.

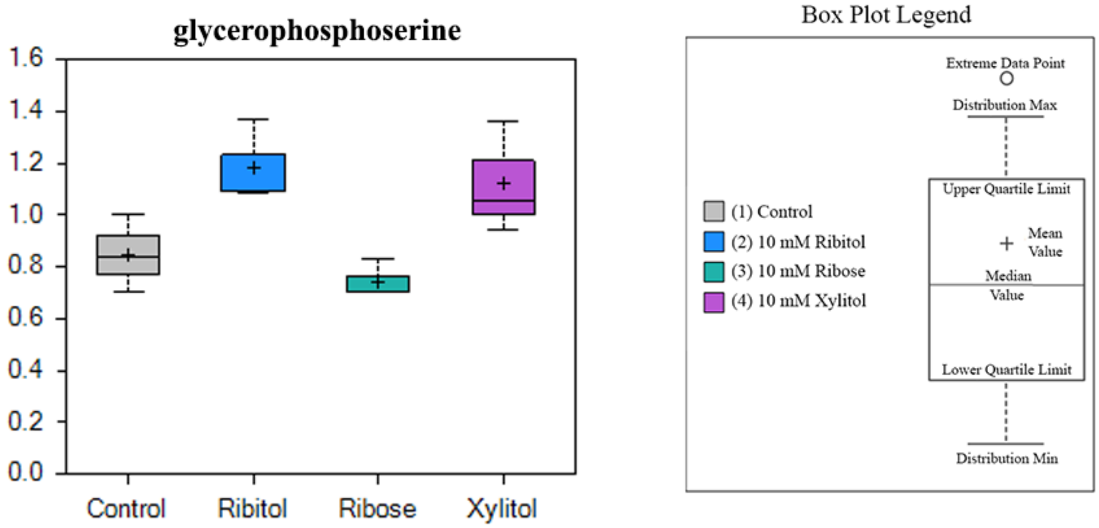

SI Figure 3: Sphingomyelins and glycerophosphoserines in cells cultured with ribitol, ribose, and xylitol supplementation.

The levels of esphingomyelins (SPHs) in the ribitol treated cells were also at the levels similar or slightly higher than that in cells treated with ribose, and significantly higher than the control. Consistent to the increased levels of serine with ribitol treatment, levels of glycerophosphoserines (GPS) were also slightly higher than the controls as shown here. The clear contrast in levels of many lipids and phospholipids between the cells treated with ribitol and ribose, is consistent with the decrease and increase in levels of citrate with the two compounds, respectively. Limited changes were observed in most of the detected lipids and phospholipids in cells treated with xylitol compared to the control. Significance denoted by $*p \leq 0.05$, $**p \leq 0.01$, $***p \leq 0.001$ within individual metabolite box plots, as determined by Welch's two-sample t-Test.

Control Avg (log2)	Ribitol Avg (log2)	Ribose Avg (log2)	Xylitol Avg (log2)	Control S.Dev	Ribitol S.Dev	Ribose S.Dev	Xylitol S.Dev	Ribitol vs Control FC	Ribose vs Control FC	Xylitol vs Control FC	Ribitol vs Control P-val	Ribose vs Control P-val	Xylitol vs Control P-val	Ribitol vs Control FDR P-val	Ribose vs Control FDR P-val	Xylitol vs Control FDR P-val	Gene Symbol
13.18	12.92	13.09	13.05	0.12	0.17	0.03	0.01	-1.19	-1.06	-1.09	**0.094**	0.7973	0.5215	0.8191	0.9717	0.9785	**ACLY**
11.1	10.75	11.35	10.88	0.09	0.13	0.07	0.05	-1.27	1.19	-1.17	**0.023**	**0.0356**	**0.094**	0.7157	0.665	0.9192	**ACO2**
14.11	14.13	14.56	14.29	0.06	0.1	0.14	0.17	1.01	1.36	1.14	0.9538	**0.0038**	0.3328	0.9961	0.5221	0.9677	**CS**
12.12	11.91	12.09	11.95	0.05	0.18	0.09	0.07	-1.15	-1.02	-1.13	**0.0466**	0.3633	0.1043	0.7666	0.8905	0.9196	**GSR**
8.53	8.64	8.81	8.88	0.09	0.17	0.11	0.07	1.08	1.21	1.27	0.2819	**0.0939**	**0.0122**	0.9015	0.7432	0.7819	**GSS**
12.56	12.36	12.15	12.44	0.04	0.07	0.06	0.19	-1.15	-1.32	-1.08	0.1764	**0.0106**	0.8822	0.8738	0.5848	0.9928	**HK1**
11.22	10.98	11.16	10.93	0.1	0.06	0.05	0.14	-1.18	-1.04	-1.22	**0.0592**	0.553	**0.0988**	0.7843	0.9342	0.9192	**IDH1**
5.6	5.64	5.87	5.83	0.09	0.08	0.2	0.15	1.03	1.2	1.17	0.4138	**0.0444**	0.173	0.9359	0.6937	0.9502	**PC**
6.1	6.21	6.28	6.03	0.2	0.11	0.11	0.06	1.08	1.14	-1.05	**0.0655**	**0.0217**	0.9064	0.7963	0.6176	0.9961	**PCK1**
7.67	8.08	7.13	8.23	0.11	0.26	0.22	0.26	1.32	-1.46	1.47	**0.015**	**0.0195**	**0.0031**	0.6681	0.6176	0.5924	**PCK2**
11.86	12.53	11.56	12.36	0.1	0.26	0.3	0.23	1.59	-1.24	1.41	**0.0056**	0.1887	**0.0095**	0.6181	0.8262	0.7728	**PHGDH**
10.16	10.13	9.8	10.1	0.09	0.11	0.17	0.05	-1.02	-1.28	-1.04	0.7591	**0.0111**	0.6891	0.9826	0.5848	0.9865	**SDHD**
9.58	9.41	9.24	9.36	0.14	0.21	0.35	0.09	-1.13	-1.26	-1.16	0.2499	**0.0144**	0.271	0.896	0.5892	0.9676	**TALDO1**

SI Table 1: Differentially expressed transcripts of metabolism-associated genes with ribitol, ribose, and xylitol media supplementation.

Illustrated here are genes which fall into those regulating glycolysis, gluconeogenesis, glutaminolysis, TCA pathways as well as GSH/GSSG levels. After aggregation of metabolically associated genes, cutoff of $p<0.10$ was used to filter the results for relation to metabolomic observations with the same treatments.

Control Avg (log2)	Ribitol Avg (log2)	Ribose Avg (log2)	Xylitol Avg (log2)	Control S.Dev	Ribitol S.Dev	Ribose S.Dev	Xylitol S.Dev	Ribitol vs Control FC	Ribose vs Control FC	Xylitol vs Control FC	Ribitol vs Control P-val	Ribose vs Control P-val	Xylitol vs Control P-val	Ribitol vs Control FDR P-val	Ribose vs Control FDR P-val	Xylitol vs Control FDR P-val	Gene Symbol
9	9.44	9.23	9.31	0.1	0.15	0.03	0.08	1.36	1.17	1.24	**0.0056**	0.1569	**0.0205**	0.6181	0.8064	0.7976	**AKTIP**
10.52	10.24	10.21	10.21	0.23	0.19	0.07	0.1	-1.21	-1.24	-1.23	**0.0168**	**0.0376**	**0.0189**	0.6724	0.6717	0.7976	**PIK3CB**
6.46	6.05	5.9	6.36	0.2	0.09	0.26	0.2	-1.33	-1.47	-1.07	**0.0207**	**0.0369**	0.7394	0.6964	0.6717	0.9865	**RASA3**
14.22	13.95	14.25	14.13	0.11	0.16	0.06	0.08	-1.21	1.02	-1.07	**0.0304**	0.9267	0.5573	0.7223	0.9924	0.9786	**GRB2**
4.3	4.75	4.78	4.53	0.15	0.29	0.43	0.08	1.37	1.4	1.18	**0.0384**	**0.0255**	0.3434	0.7441	0.6324	0.968	**HIF3A**
4.18	3.87	4.05	4.22	0.22	0.07	0.2	0.03	-1.24	-1.09	1.02	**0.0409**	0.2671	0.7155	0.746	0.8654	0.9865	**RASSF2**
3.09	3.28	3.13	3.02	0.08	0.13	0.18	0.09	1.14	1.03	-1.05	**0.0418**	0.6899	0.3837	0.746	0.9554	0.9699	**TP53TG3B**
3.09	3.28	3.13	3.02	0.08	0.13	0.18	0.09	1.14	1.03	-1.05	**0.0418**	0.6899	0.3837	0.746	0.9554	0.9699	**TP53TG3B**
3.09	3.28	3.13	3.02	0.08	0.13	0.18	0.09	1.14	1.03	-1.05	**0.0418**	0.6899	0.3837	0.746	0.9554	0.9699	**TP53TG3B**
9.91	10.08	10.02	9.97	0.12	0.12	0.05	0.04	1.13	1.08	1.05	**0.048**	0.2378	0.5373	0.7666	0.8486	0.9785	**PIKFYVE**
10.34	10.16	10.13	10.06	0.04	0.11	0.08	0.07	-1.13	-1.16	-1.22	**0.0488**	0.0861	**0.0229**	0.7666	0.7311	0.8145	**MAP2K1**
3.66	3.32	3.61	3.3	0.15	0.12	0.17	0.15	-1.26	-1.03	-1.28	**0.024**	0.7307	**0.0341**	0.7193	0.9628	0.861	**RASL12**
3.78	5.03	4.62	4.05	0.58	0.68	0.27	0.38	2.38	1.78	1.2	**0.0339**	0.0543	0.5829	0.7344	0.7096	0.9798	**KRAS**
6.69	6.42	6.35	6.76	0.02	0.34	0.2	0.22	-1.2	-1.26	1.05	**0.0428**	0.0542	0.7719	0.7505	0.7096	0.9865	**PIK3CD**
5.78	6.06	5.93	5.94	0.23	0.06	0.11	0.13	1.21	1.11	1.11	**0.0477**	0.3727	0.089	0.7666	0.8927	0.9178	**CDKN2C**
6.91	7.44	7.03	6.91	0.33	0.4	0.11	0.4	1.44	1.09	1	**0.0514**	0.314	0.5367	0.7666	0.8782	0.9785	**AKT1S1**
9.97	9.65	9.58	9.85	0.2	0.12	0.23	0.17	-1.25	-1.31	-1.08	**0.0566**	**0.027**	0.2143	0.778	0.6365	0.9643	**CBLB**
11.08	10.82	10.89	10.84	0.15	0.11	0.14	0.06	-1.2	-1.14	-1.18	**0.0576**	0.1426	0.0952	0.7781	0.7927	0.9192	**MYCBP**
8.97	9.18	9.2	9.16	0.07	0.2	0.15	0.09	1.16	1.18	1.14	**0.0614**	**0.0491**	0.0991	0.7884	0.7052	0.9192	**EPS8**
8.46	8.07	8.39	8.3	0.19	0.32	0.21	0.13	-1.31	-1.05	-1.12	**0.0798**	0.5065	0.6394	0.8143	0.9256	0.9832	**LAMTOR1**
3.39	3.25	3.53	3.52	0.32	0.25	0.15	0.14	-1.11	1.1	1.09	**0.08**	0.9368	0.7616	0.8143	0.9931	0.9865	**RASA3**
5.14	5.62	5.24	5.21	0.16	0.31	0.22	0.05	1.4	1.07	1.05	**0.0896**	0.8744	0.6468	0.8166	0.983	0.9835	**HBEGF**
12.37	12.53	12.8	12.52	0.09	0.16	0.1	0.06	1.11	1.34	1.11	**0.096**	**0.002**	0.1229	0.8206	0.4871	0.9262	**TP53**

SI Table 2: Differentially expressed transcripts of oncology- and RAS-associated genes with ribitol, ribose, and xylitol media supplementation.

Expression status of genes known to be related to RAS related cell proliferation and growth inhibition. These genes can be divided into two groups, one with oncogenic or cell proliferation potential and the other considered as tumor suppressor. Genes with oncogenic potential were either upregulated (AKTIP, 1.36 fold; HIF3A, 1.37 fold; PIKFYVE, 1.13 fold), or downregulated (PIK3CB, -1.21 fold; GRB2, -1.21 fold; MAP2K1, -1.13 fold) in the cells with ribitol treatment as shown here. Similarly, the identified tumor suppressors were also either up-regulated (TP53TG3B; TP53TG3C, 1.14 fold) or down-regulated (RASA3, -1.33 fold; RASSF2, -1.24 fold) It is therefore probable that alteration in expression of these genes reflects slightly improved cell growth by ribitol, rather than an oncogenic potential. After aggregation of oncologically-associated genes, cutoff of $p<0.05$ was used to filter the results for relation to metabolomic observations with the same treatments.

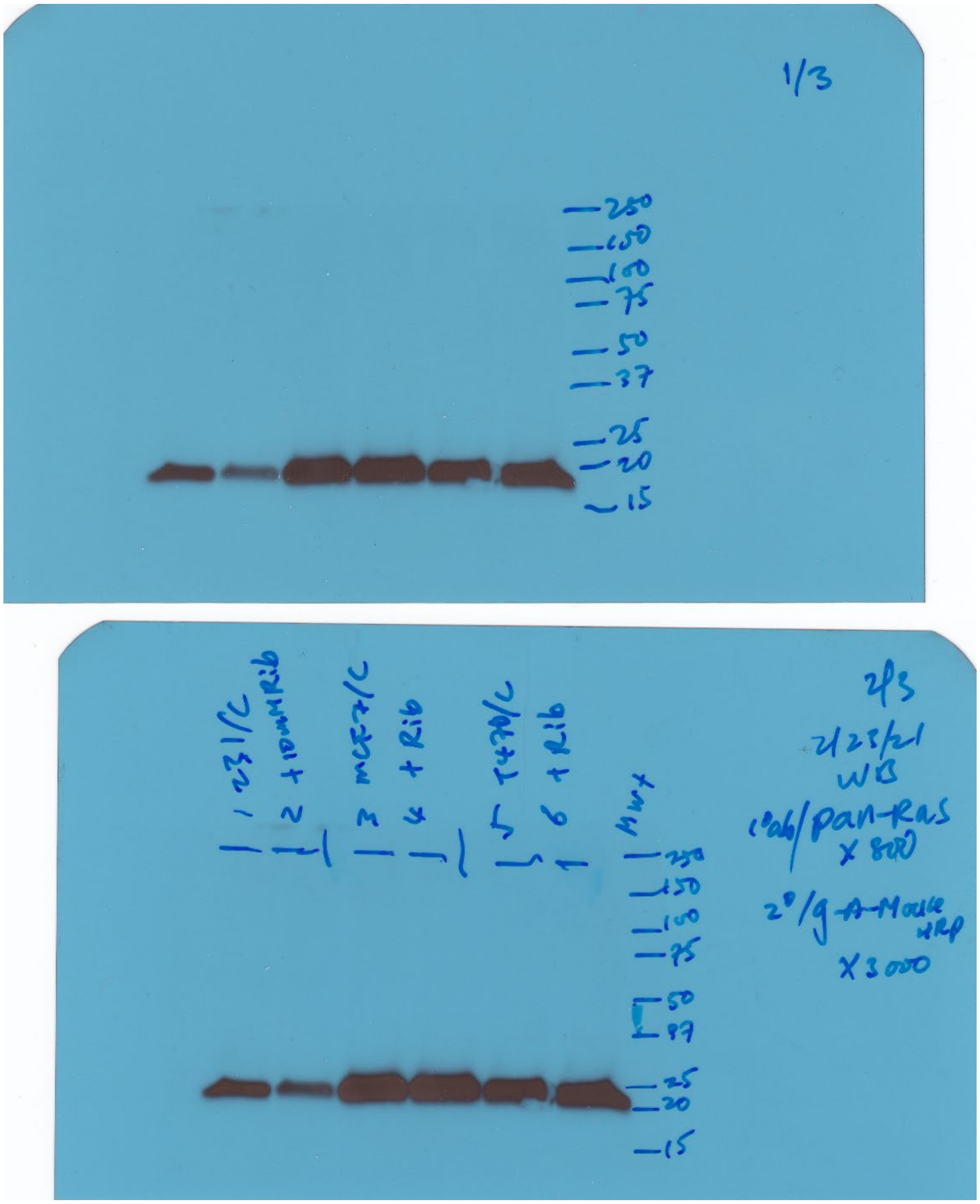

SI Figure 4: Raw image of KRAS western blot.

Supplementary Data File 1: Metabolomic Data

Supplementary Data File 2: Transcriptomic Data
